# IGAP-integrative genome analysis pipeline reveals new gene regulatory model associated with nonspecific TF-DNA binding affinity

**DOI:** 10.1016/j.csbj.2020.05.024

**Published:** 2020-06-02

**Authors:** Alireza Sahaf Naeini, Amna Farooq, Magnar Bjørås, Junbai Wang

**Affiliations:** a Department of Pathology, Oslo University Hospital - Norwegian Radium Hospital, Oslo, Norway; b Institute for Cancer Research and Molecular Medicine, Norwegian University of Science and Technology, Trondheim, Norway; c Department of Microbiology, Oslo University Hospital, Oslo, Norway

**Keywords:** Transcription factor binding, DNA sequence, Chromosomal interaction, Bioinformaitcs, Genome regulation

## Abstract

The human genome is regulated in a multi-dimensional way. While biophysical factors like Non-specific Transcription factor Binding Affinity (nTBA) act at DNA sequence level, other factors act above sequence levels such as histone modifications and 3-D chromosomal interactions. This multidimensionality of regulation requires many of these factors for a proper understanding of the regulatory landscape of the human genome. Here, we propose a new biophysical model for estimating nTBA. Integration of nTBA with chromatin modifications and chromosomal interactions, using a new Integrative Genome Analysis Pipeline (IGAP), reveals additive effects of nTBA to regulatory DNA sequences and identifies three types of genomic zones in the human genome (Inactive Genomic Zones, Poised Genomic Zones, and Active Genomic Zones). It also unveils a novel long distance gene regulatory model: chromosomal interactions reduce the physical distance between the high occupancy target (HOT) regions that results in high nTBA to DNA in the area, which in turn attract TFs to such regions with higher binding potential. These findings will help to elucidate the three-dimensional diffusion process that TFs use during their search for the right targets.

## Graphical abstract

**Figure:**
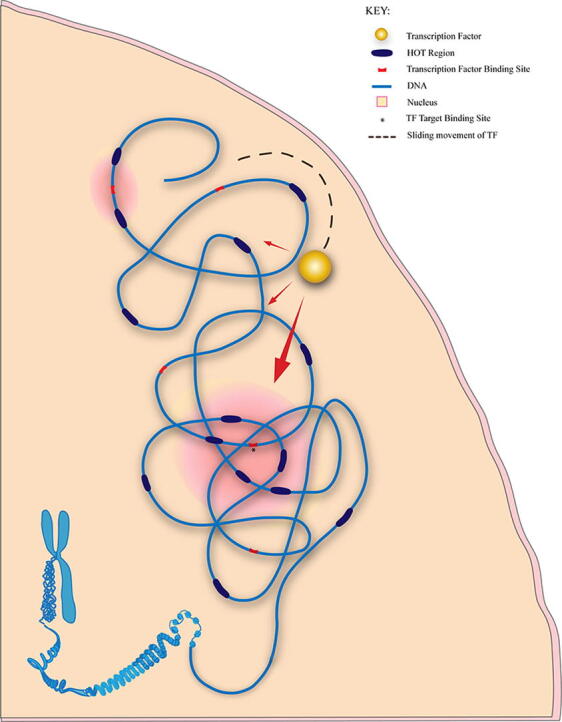

## 1 Introduction

Unraveling the regulatory complexity of the genome is a challenging task. With ever-improving high throughput technologies, there is a multitude of data sets at our disposal, such as gene expression profiles, genetic variants and epigenetic profiles. Furthermore, the spatial organization of chromatin has been determined in many cell lines and conditions [1], [2], [3], [4]. Nonspecific protein-DNA interactions [5] and chromatin modifications (e.g., histone modifications, nucleosome occupancy, and chromosome interactions) are not sparsely distributed in the genome, they in fact, participate in gene regulation [5], [6]. However, the relationship between the various chromatin features remains to be investigated. In order to obtain a comprehensive view, a variety of available information needs to be combined as no single approach is sufficient to elucidate the regulatory landscape of the genome.

Genome regulation is a multileveled phenomenon initiated by specific and non-specific biophysical interactions between transcription factors (TF) and DNA [7], [8]. For instance, Transcription Start Sites (TSS) serve as assembly points for transcription initiation complexes which are vital elements of transcription regulation. Enhancers serve as a platform for recruiting sequence-specific TFs and co-activators, thereby regulating the assembly of the active transcription machinery. Notably, several studies [9], [10] show that TF binding sites alone are not sufficient for TF binding to occur, suggesting that other factors like non-specific TF binding affinity (nTBA) might play an important role. Conventionally, low-affinity and non-specific genomic binding events, though frequent, were considered functionally irrelevant. However, recent studies have shown that low affinity TF binding is functional [11].

Apart from TSS, enhancers, and super-enhancers (SE) or clustered enhancers [12], high occupancy target (HOT) regions exhibit an exceptionally high frequency of TF-DNA binding events. Although HOT regions attract hundreds of different TFs [13], these regions do not contain an equal number of canonical motifs [14]. There are different assumptions regarding their role in genome regulation [15]. For instance, it is assumed that HOT regions play a role in combinatorial interaction of TF, where only a few of them bind to HOT regions [16]. However, the stability of a protein complex comprising hundreds of TFs and co-factors supported by a few canonical binding sites seems questionable. Hence, the interplay between HOT regions and TFs merits further investigations.

Epigenetic modifications and chromatin structure are important layers of genome regulation. For instance, histone modifications modulate DNA accessibility for TFs [17]. Likewise, many studies have demonstrated a significant role of chromosomal interactions in genome regulation [18], [19]. Techniques like Chromosome Conformation Capture (3C) assays and imaging studies have proved that distal regulatory regions like enhancers come into physical proximity of target promoters by looping out intermediate sequences [17], [20], [21], [22]. The mechanism and dynamics behind these long-range interactions remain unknown. Similarly, high-resolution Hi-C studies have suggested that chromosome topology exerts a significant impact on enhancer-promoter communication and resulting gene expression [21]. Intermixing regions of chromosomes are enriched for TF binding sites and other genomic markers [18]. It is believed, integrating other genomic data can identify key regulatory zones in genomes, which can be useful in searching for functional genomic elements and interactions.

There is a long history of applying the biophysical theory of protein-DNA interactions on computational gene regulation studies. The field is being developed rapidly from the pioneer theoretical work of von Hippel P and Berg OG [23] to more recent works of TF binding sites discovery [24], [25], predicting TF binding affinity to DNA [26], [27], and inferring conditional dependent TF binding energies [28], [29]. In our previous studies, we have developed biophysical models [1], [29], [30] to distinguish direct versus indirect protein-DNA interactions from ChIP-seq experiments [31], to rank regulatory mutations in disease by using TF differential binding affinity (*dbA*) [32], and to find functional regulatory effects of mutations in cancer patient cohorts [33], by integrating genome-wide sequencing data with diverse information (e.g. the shifted differential binding affinity (δdbA) and the gene expression profiles). Inspired by a work [34] of Berg OG et al., where models and theories for how TFs localize to the DNA target sites were proposed, we recently became interested in three-dimensional diffusion processes of regulatory proteins for the search of the true target sites. These processes may be simplified into a two-step binding mechanism: target searching and target binding. First, TFs are sliding nonspecifically on DNA sequences searching for the targets, and then hopping/sliding to specific (target) sites for target binding when certain conditions are triggered. There is experimental evidence of TFs pausing at sites resembling their recognition sites and in some cases, binding nonspecifically to generic DNA sequences [35], [36]. Thus, it can be inferred that every regulatory protein (or TF) has some affinity for nonspecific DNA in addition to the specific target site. Hence, it is tempting to study the relationship between the nTBA to DNA and the other genomic features (e.g., histone modifications and nucleosome occupancy) at Transcription Start Site (TSS), gene centers (±500 bp to the center of gene transcribed region), enhancers and High Occupancy Target (HOT) regions. Thus, a new biophysical model for computing nTBA on DNA sequences, and a pipeline to integrate such new sequence features with a variety of genomic information, are implemented in Python. The integrated data analysis pipeline is biologically motivated and statistically justified, which can be used to classify genomes into activity based zones and reveal the regulatory role of otherwise blur genomic elements e.g., HOT regions.

## 2 Material and methods

### 2.1 Data collection

#### 2.1.1 Human genome annotation

Gene and transcription start site (TSS) positions are obtained from UCSC human genome hg19 RefSeq database. Predicted human enhancer regions are acquired from EnhancerAtlas database [37], where we selected ∼ 465399 enhancers from thirty tissues for the current study. *In silico* predicted super-enhancers (SE) and hub-enhancers (HE) in K562 (∼843 SE, ∼444 HE) and GM12878 (∼834 SE, ∼606 HE) cell lines are obtained from a previous publication [12]. Predicted HOT (High-occupancy TF target) regions in human genome (e.g., ∼71583 HOT in any of the human contexts) are downloaded from earlier work [1] of the ENCODE project. A combined segmentation of the human genome, generated by two machine-learning-based methods ChromHMM [38] and Segway [39], are obtained from a previous publication [40]. These chromatin state segmentations were produced on basis of chromatin features in the two cell lines (e.g., histone modification and nucleosome occupancy in K562 and GM12878, respectively). The combined segmentation from the two methods uses only seven chromatin states to segment the genome in functional regions.

#### 2.1.2 Human TF ChIP-seq data

Two input datasets for human TF ChIP-seq were used. The first set of called peaks of ChIP-seq experiments from human CTCF, NRSF, STAT1, ERα (ER1), and SPIB were downloaded from previous publications [31], [41], [42], [43]. These ChIP-seq experiments were conducted on various cell lines; CD4+ T (helper cells cell line), Jurkat T lymphoblast (acute lymphoblastic leukemia cell line), Hela S3 (cervical cancer cell line), MCF-7 (breast cancer cell line), and HBL1 (B cell lymphoma cell line). To investigate reproducibility of results, another input data set was collected from ENCODE (GEO accession numbers in Supplementary Table 1). For the second input dataset, called peaks were downloaded for two different cell lines for each of the five TFs. For both ERα and SPIB, the predicted direct and indirect TF targets were obtained from previous work [31], where a biophysical model was used to distinguish type I (direct) versus type II (indirect) TF binding sites using ChIP-seq experiments and the partial predictions were verified by Electrophoretic mobility shift assay (EMSA).

#### 2.1.3 Human histone modification and expression data

Histone modification marks (H3K27ac, H3K4me1, H3K4me3, H3K27me3, H3K9me3), RNA Polymerase II, CTCF, and DNase-seq data sets in basal MCF-7, K562 (myelogenous leukemia cell line), and GM12878 (lymphoblastoid cell line) cell lines were obtained from the ENCODE Project [3]. The majority of cell lines lacked data set for one or more of the genomic marks of interest for this project. Hence, three cell lines that were most established and had data available for all the selected genomic marks: one normal cell line (GM12878) and two cancer cell lines (MCF-7, K562) were chosen. The aligned (hg19) BAM files were downloaded from the University of California at Santa Cruz (UCSC) genome browser database [44].

#### 2.1.4 Hi-C data

Intra-chromosomal interaction contact matrix for basal MCF-7, K562, and GM12878 cells, were obtained from earlier publications [45], [46]. The GEO accession numbers of the datasets are GSE66733 and GSE63525 [47].

### 2.2 Data processing

Gene, TSS, enhancer, super-enhancer, hub-enhancer, seven merged chromatin states, and HOT region data was subjected to preprocessing for the sake of introducing uniformity in data. All elements shorter than 100 bp were removed. For sake of unanimity of nTBA calculation, a 1000 bp window centered on the median of each element was extracted. Any duplicate windows were deleted. For each BAM file of human histone modification and expression data, BEDTools [48] were first used to estimate the read counts in a 100 bp window size. Then, the raw read counts were normalized across all markers. The normalization method is similar to a previous publication [49]. If multiple ChIP-seq experiments represent the same marker in the same cell line, then an average of their normalized read counts was used. All normalized reads counts were log-transformed and converted to Z-scores before performing further data analysis. Hi-C interaction data were transformed to Z-scores [50] before studying the intra-chromosomal interactions. Generation of an intra-chromosomal interaction contact matrix has been described earlier [31], [51]. Briefly, binned data were first subjected to normalization and transformation to Z-scores, the distributions of intra-chromosomal interaction frequencies for the three cell lines were then examined at 250 kb resolution, respectively. More specifically, for every 250 kb of chromosome regions, the number of interactions between each chromosome region and the rest of the chromosome regions were counted. The count matrix of intra-chromosomal interactions was normalized by either the KR matrix balance method (K562 and GM12878 cells) [52] or the iterative correction and eigenvector decomposition (ICE) [53] method (MCF7 cell) in the original work [45], [46]. Then Z-scores of normalized intra-chromosomal interaction matrices were generated, from which the chromosome-specific intra-chromosomal contact heat maps and the intra-chromosomal interaction frequency in the binned regions were obtained.

#### 2.2.1 A new biophysical model to estimate nonspecific TF binding affinity to DNA

**Fig. 1:**
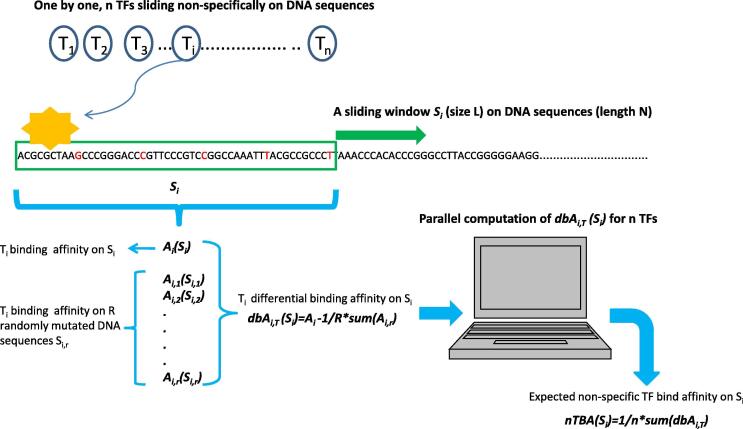
Illustration of calculations of non-specific TF binding affinity on DNA sequences. A cartoon diagram is used to illustrate the proposed new biophysical model for estimating non-specific TF binding affinity (nTBA) on DNA sequences.

By considering both the work of Berg OG et al., [34] and our previous studies of TF binding affinity calculations [31], [32], we have developed a new biophysical model to calculate non-specific TF Binding Affinity (nTBA) to DNA. In this calculation, a sliding window analysis is adopted to compute nTBA on DNA sequences. Here the number of TFs and the number of target sites are represented by the number of available position weight matrices (PWM) in a collection and the number of windows sliding on the DNA sequences, respectively. Briefly, a window (or a non-specific target site *S_i_*) with size L (i.e. 50 bp) and step size 5 bp is sliding on the DNA sequences. For each sliding window, a computation of nTBA at *S_i_* is illustrated by Fig. 1, where we assume *n* TFs (i.e. *n* = 1772 PWMS from earlier work [32]) slide one-by-one on DNA sequences non-specifically. For every TF, the number of non-specific target sites is the total number of windows that are sliding on a DNA sequence with length *N*. If the window size is *L* and the step size is *st*, then the total number of windows/target sites are

$$k=\frac{\left(N-\frac{L}{2}\right)-L/2}{\mathrm{st}}$$

Here, the estimated nTBA is assigned to the center of a window bin. There are many TFs (*n*) which can walk randomly and interact with many different non-specific target sites (i.e. *k* sliding windows on DNA sequences) in a nucleus, before reaching the true target site. This is a many-body problem where many TFs interact with many putative target sites and is difficult to solve. Nevertheless, such a many-body system can be simplified to a one-body system by applying Mean Field Theory (MFT) from statistical physics [54]. In the one body system, an effective *dbA* (or expected nTBA) is computed at each arbitrary target binding site (or a sliding window *S_i_*) to DNA.

There are two hypotheses in the calculation of nTBA: First, all TFs (n) are assumed to bind with equal prior probability to all available target sites (k) on DNA sequences; Second, both the adjacent sliding windows (adjacent target sites) on DNA sequences and the TFs in a nucleus are independent of each other, implicating that they do not affect each other when a TF is sliding on a DNA sequence. These assumptions are borrowed from MFT and the theoretical foundation is similar to a previous work [55], which allow us to replace the interactions of many TFs (e.g., n TFs) to a target binding site (i.e. a sliding window *S_i_* in Fig. 1) with an average/effective interaction [56]. Thus, the complex many-body system is reduced to an effective one-body system, where an effective *dbA* for a sliding window *S_i_* (or an average of *dbAs* from many TFs at a target *S_i_*) is obtained. This effective *dbA* can be seen as an expected nTBA to the target sequence *S_i_*. In this way, a new DNA sequence feature (expected nTBA) is computed in a whole chromosome by sliding a window on the DNA sequences.

In Fig. 1, there is an illustration of the computation of nTBA in a sliding window *S_i_*. A more detailed description is provided here. Based on a biophysical theory of protein-DNA interactions [29], [57], a Femi-Dirac form of protein binding probability is used to estimate protein-DNA binding affinity that depends on the chemical potential (or the protein concentration), $P\left(S\right)=\frac{1}{1+exp(E*S-\mu )}$, where *E* represents PWM or a protein binding energy matrix (PBEM), *μ* is the chemical potential or the concentration of proteins in a solution, and *P* is the probability of a DNA sequence *S* to be bound by a TF. However, in cases where protein-DNA interactions do not depend on the chemical potential (*μ* = *0*), or the protein concentration is very low, we use a Maxwell-Boltzmann protein binding function P(S)≈exp(-E∗S) [25] to calculate the TF binding affinity. The binding affinity of a TF on a sliding window is computed by function $A_{i}=p\sum_{l=1}^{L-m+1}P_{i,l}\left(S_{i}\right)$, i=1⋯N-L+stepsize, where S_i_ represents DNA sequences in the sliding window, L is the window size (i.e. L = 50 bp), *N* is the length of DNA sequences, *m* is the length of TF binding site (e.g., the length of a PWM), *p* is a prior probability of a TF binds to target sequence $S_{i}$. In this study, we assume all $S_{i}$ have equal probability of being bound by a TF (p=1). In the calculation, *P_i,l_*(*S_i_*) is a protein-DNA binding probability function that depends on the chemical potential, which uses either Femi-Dirac or Maxwell-Boltzmann form of protein binding probability.

For a TF sliding nonspecifically on the target sequence $S_{i}$, we first compute its differential binding affinity ${\mathrm{dbA}}_{i,T}\left(S_{i}\right)$ at sequence $S_{i}$, as ${\mathrm{dbA}}_{i,T}=A_{i}-A_{i,R}$.The calculation of $A_{i}$ is defined in the previous section, $A_{i,R}$ is the average of TF binding affinity for randomly mutated DNA sequence $S_{i,r}$, $A_{i,R}=\frac{1}{R}\sum_{r=1}^{R}p\sum_{l=1}^{N-m+1}P_{i,l}\left(S_{i,r}\right)$, *R* is the number of random shuffling of DNA sequence *S_i_*. In this way, ${\mathrm{dbA}}_{i,T}$ removes potential bias from the local DNA sequences that allows us to compare the TF binding affinity genome-widely [32]. At the same target site or a sliding window $S_{i}$, we repeat the ${\mathrm{dbA}}_{i,T}$ calculation for all available TFs (i.e. ∼1772 PWMs in the collection). Later, an average of ${\mathrm{dbA}}_{i,T}$ calculated for all available TFs is used to represent the expected nTBA to DNA $S_{i}$: for example, $\mathrm{nTBA}\left(S_{i}\right)=\frac{1}{n}\sum_{T=1}^{n}{\mathrm{dbA}}_{i,T}$, where *n* is the number of TFs considered in the calculation. Finally, the nTBA for all the target sites on DNA sequences is estimated by using the sliding window approach, where the estimated $\mathrm{nTBA}(S_{i})$ of each target site is assigned to the center of a sliding window. In order to reduce the overall computational burden, the calculations of $\mathrm{nTBA}(S_{i})$ at target sequence $S_{i}$ of multiple TFs are split to multiple computer processes and are run in parallel. A simple diagram of the nTBA calculation in sliding window analysis is shown in Fig. 1. Consequently, the new DNA sequence feature – nTBA is computed genome-wide, which can be integrated with other epigenomic modifications to investigate the gene regulation.

#### 2.2.2 Enrichment test of epigenomic modifications in pair-wise intra-chromosomal interactions

For every genomic marker or an epigenomic modification (e.g. histone modification, Pol II expression, CTCF binding, DNase occupancy, or nonspecific TF binding affinity), we compared its enrichment in a Hi-C detected pair-wise intra-chromosomal interaction to that in an average of randomly selected (e.g., 100 times) pair-wise intra-chromosomal interactions, by using Rank-Sum test. In this work, the detected intra-chromosomal interaction (e.g., Z-scores >0 in the interaction contact matrix) means the interaction is more frequent than the average of genome-wide interactions (e.g., red color in intra-chromosomal interaction heat-map). Results of the enrichment tests are shown in a heat-map, where the blue and yellow color represent the negative and positive Z-values from the Rank-Sum test, respectively. For example, in every 250 Kb interaction region (a genomic window bin), only markers located at ±500 bp from TSS/HOT center are considered. If there are multiple TSS or HOT regions located in the same bin (250 Kb), then an average of them will be used in the pair-wise enrichment test. However, for nonspecific TF binding affinity, if there are multiple TSS or HOT regions (e.g., ±500 bp from the TSS/HOT center) in the same window bin, then the sum of their nTBA will be used in the enrichment test. In this way, we are able to study possible links between the additive effect of nTBA to HOT/TSS regions and the enrichment of other epigenomic modifications (e.g., H3K27ac, H3K4me1, H3K4me3, H3K27me3, H3K9me3, etc.) in TSS and HOT regions of pair-wise intra-chromosomal interactions.

#### 2.2.3 A comparison between the frequency of intra-chromosomal interactions and the frequency of highly enriched epigenomic modifications in genomic window bins

The generation of intra-chromosomal interaction frequencies is similar to a previous publication [31] which was examined using a 250 Kb resolution genomic window bin. Briefly, for each 250 Kb bin of chromosome regions, the number ($N_{\mathrm{vi}}$) of detected interactions (i.e., Z-scores >0) between each chromosome region and the rest of the regions in a chromosome was counted. Simultaneously, the number ${(N}_{\mathrm{ei}})$ of its high enrichment (e.g. Rank-Sum test Z-value ≥3 [32]) in the detected intra-interactions was also recorded for each marker (or epigenomic modification). The intra-chromosomal interaction frequency $F_{i}$ of the region was then calculated as the counted number ($N_{\mathrm{vi}}$) of interactions in the region divided by the total number ${(N}_{t})$ of genomic window bins in a chromosome$F_{i}$ = $N_{\mathrm{vi}}/N_{t}$. In a genomic window bin, the frequency ${(F}_{\mathrm{he}})$ of a highly enriched epigenomic modification in intra-chromosomal interactions is$F_{\mathrm{he}}$ = $N_{\mathrm{ei}}/N_{t}$. With a 250 Kb resolution genomic window bins, $N_{t}$ equals 325 and 253 for human chromosomes 17 and 20, respectively. This study was conducted on human chromosome 17 and 20 initially as a pilot study. After optimization of the complete pipeline on chromosome 17 and 20, we later expanded it to the whole human genome (except chromosome Y, because of the unavailability of data).

In order to estimate an optimal number of clusters in genomic window bins in a chromosome (e.g. ∼ 325 of 250 kb bins in chromosome 17), a stress function [58] was used based on the frequencies ${(F}_{\mathrm{he}})$ of the highly enriched epigenomic modifications or genomic markers (e.g. nTBA, nucleosome occupancy, Pol2 expression, enhancer/promoter histone markers, and CTCF binding) in the bins. After determining the cluster size, a cluster number is assigned to each chromosome. For this aim, initially, k-means [59] algorithm is used to cluster $F_{\mathrm{he}}$ in window bins, then an inverse of Euclidean distance between each region and centroid of each cluster is calculated. Scores of each region are divided by their sum, which can be considered as a probability. Then, a Gaussian mixture model [59] is fitted based on the same number of components as that of clusters to the $F_{\mathrm{he}}$in window bins, and a set of probabilities for each region related to different components are obtained. Finally, based on the weighted sum approach, the highest probability of a cluster is assigned to the region. Subsequently, the $F_{\mathrm{he}}$ of all epigenomic modifications are shown in the heat maps, where the genomic window bins are classified by the aforementioned method. The frequencies $F_{i}$ of intra-chromosomal interactions in genomic window bins are also plotted using scatter and box plot, where colors are labeled according to the clusters. In this way, a glance at the clustering of $F_{\mathrm{he}}$ heat-map and the $F_{i}$ plots may reveal a relationship of frequencies between the intra-chromosomal interactions and the highly enriched epigenomic modification in genomic window bins.

#### 2.2.4 Generation of an integrative genome analysis pipeline (IGAP)

Aforementioned integrative data analysis is implemented in a user-friendly python package (https://igap-pipeline.github.io/igap/): Integrative Genome Analysis Pipeline (IGAP). There are mainly five major functions in IGAP:

1. Compute nonspecific TF binding affinity (nTBA) for DNA sequences.
2. Obtain nTBA values for predefined genomic regions (e.g., TSS and enhancer).
3. Obtain epigenetic modification or other genomic marks (e.g., nucleosome occupancy) for the genomic regions.
4. Obtain chromosomal interaction frequency (e.g., Hi-C data) for the genomic regions.
5. Clustering of regions based on generated feature vectors from the previous four functions.

**Fig. 2:**
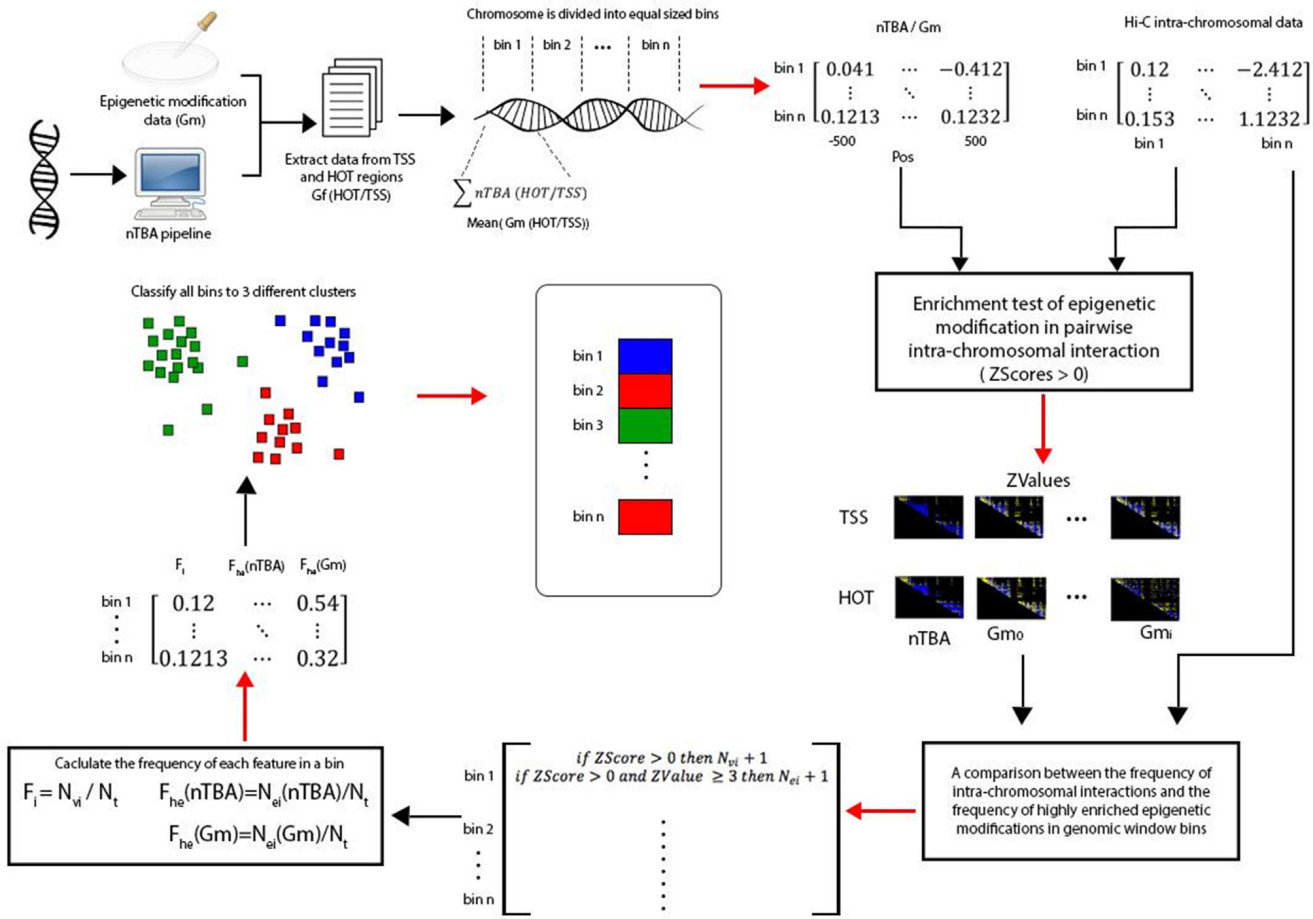
Detailed work flow of integrative genome analysis pipeline. The initial phase of pipeline constitutes of the calculations of nTBA for any desired genomic region. The given input sequence is divided into equal sized (250 kb) bins. Genomic features belonging to each bin are mapped bin wise in the form of a frequency matrix. All the genomic markers/features data is integrated to finally draw out Inactive (red), Poised (green) and Active (blue) Zones of the genome. (For interpretation of the references to color in this figure legend, the reader is referred to the web version of this article.)

More detailed description of these functions and usage of IGAP pipeline please refer to IGAP web supplementary, where there are both user manual and sample demos for the package. An overview of IGAP is illustrated in Fig. 2. IGAP first calculates nTBA for a given DNA sequence (e.g., chromosome 17). Then it considers other genomic features from either experimental observations or computational simulations. After all input datasets are ready, genomic regions of interest are extracted (e.g. HOT and TSS). Here, the input DNA sequence is divided into equal sized bins, where values of genomic features from each bin are calculated. After integrating Hi-C intra-interactions, the frequency of enrichment of features in a bin is calculated (method Sections 2.2.2 and 2.2.3). In the end, feature vectors of input genomic features are generated, which can be used to classify input genomic window bins to different regions such as type I/II/III bins (or Active/Poised/Inactive Genomic Zones). IGAP can be a useful tool for multiple studies providing new insight about genome regulation.

### 2.3 Transcription factor binding site enrichment in gene promoter region

For genes associated with our predicted three core types of window bins, TF binding sites enrichment test was performed on the gene promoter regions, respectively. One is a default method from DAVID tool [60], which utilizes conserved transcription factor binding sites from three species human/mouse/rat (TFBS Conserved Sites track in UCSC Genome Browser) to predict over-represented TF binding sites in gene promoters. In addition, we applied two other methods Pscan [61] and PASTAA [62] for the same purpose. Pscan is similar to CLOVER algorithm [63], which tries to find enriched TF binding sites in a cluster of DNA sequences via statistical over-representation (e.g., Z-test). PASTAA computes binding affinity of TF to gene promoters based on a biophysical model and then applies hypergeometric test to evaluate TF-gene cluster associations. Here, Pscan and PASTAA use motif matrix from the JASPAR and TRANSFAC database, respectively, to scan gene promoters (e.g., −200 bp to TSS).

## 3 Results

### 3.1 Non-specific TF binding affinity at selected genome regions

To study the contribution of non-specific TF binding affinity (nTBA) to DNA in the human genome, ∼200 called peaks from five human TF ChIP-seq experiments (e.g., CTCF, ER1, NRSF, SPIB and STAT1) were randomly selected. Interestingly, the peak of total nTBA from the 200 binding sites is always found near the center of ChIP-seq called peaks for all TFs (Supplementary Fig. 1a), suggesting that nTBA may have an impact on genome regulation in addition to the direct recognition of TFs to regulatory DNA sequences. Nevertheless, decreased nTBA to DNA is followed by an increase in TF concentration and nTBA almost disappears at high TF concentrations. For example, nTBA to DNA is almost zero when the chemical potential approaches −13, −15, −18, and −20, respectively. Thus, the effect of nTBA to DNA may only be significant from low to median TF concentrations (e.g. µ between 0 and −10). The same results were observed when two sets of ∼ 200 and 500 ChIP-seq called peaks (Supplementary Fig. 1b and 1c respectively) were randomly selected for the same five TFs in ENCODE, from two different cell lines for each TF (STable 1).

**Fig. 3:**
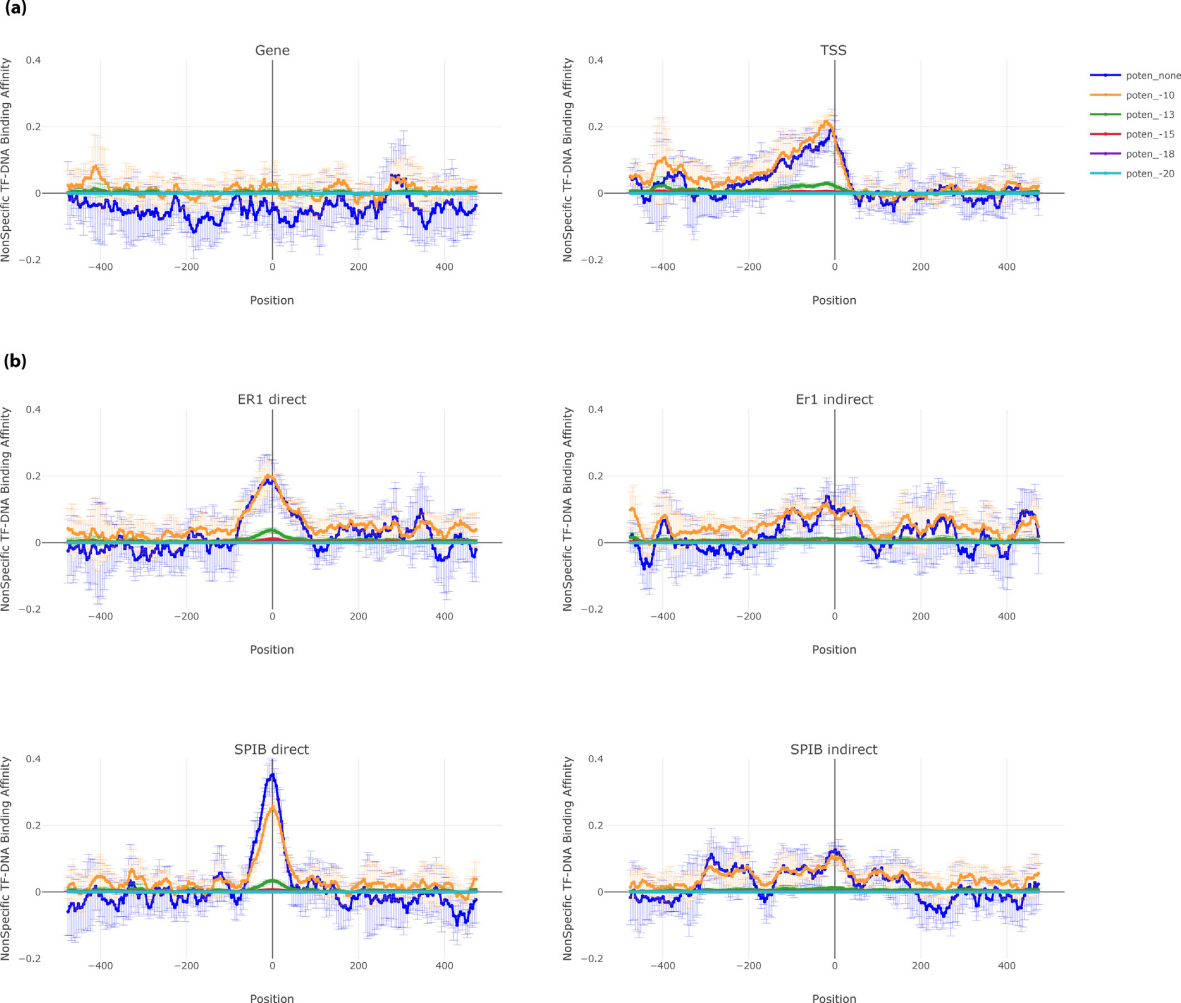
Distribution of nonspecific TF binding affinity at human genes, transcription start sites (TSS) and direct TF binding sites versus indirect TF binding sites. In panel a) For genes and TSS, we plotted the distribution of nonspecific TF binding affinity at ±500 bp centered on either gene centers or TSS. The mean and the standard deviation of the total nonspecific binding affinities from five times randomly selected genes or TSS (∼200 in each) are shown in the figure, where 0 represents either the center of a gene or TSS. In panel b), for two TFs (ESR1, and SPIB), we plotted the distribution of nonspecific TF binding affinity at ±500 bp centered in called peaks of either the direct TF binding site or the indirect TF binding one. The mean and the standard deviation of total nonspecific binding affinities from five times randomly selected peaks (e.g., ∼200 peaks in each selection) are shown in the figure, where 0 represents the center of called peaks. In both panels, predictions from different chemical potentials (or TF concentrations) are shown on different colors, respectively.

Subsequently, the same analysis is repeated in gene center and other regulatory regions such as TSS (Fig. 3a) and enhancers (Supplementary Fig. 2). These results also indicate that nTBA plays a role in addition to specific TF-binding to regulatory DNA sequences. For example, in the high chemical potential (e.g., µ equals −13, −15, −18, or −20), the pattern of nTBA is very similar among the gene centers, TSS and enhancers (e.g., close to zero). However, from low to median chemical potential (e.g., µ equals 0 or −10), the distributions of nTBA are different in different regions. For instance, the total nTBA of 200 randomly selected sites oscillates between 0 and −0.1, has a clear narrow peak, and oscillates towards a positive value 0.1 in genes (±500 bp to gene center), TSS regions (±500 bp to TSS), and tissue-specific human enhancers (e.g., ±500 bp to the enhancer center), respectively. Thus, it appears that the additive effect of nTBA to DNA is enriched in gene regulatory regions (e.g., TF binding site, TSS, or enhancer), but nearly absent in transcribed gene body or non-regulatory regions, consequently, there is an anti-correlation between nTBA to DNA and the TF concentration. Therefore, the nTBA estimated from low to median TF concentrations (e.g., µ equals 0 and −10) are appropriate for computational studies of gene regulation.

### 3.2 Non-specific TF binding affinity at direct/indirect TF target sites and HOT regions

Since the effect of nTBA to DNA differs between gene regulatory regions (e.g., TF binding sites, TSS, and enhancer) and the gene transcribed regions (e.g., gene center), it would be interesting to explore nTBA at direct/indirect TF binding target sites. Thus, five sets of putative direct/indirect TF binding sites for ER1 and SPIB (e.g. each has 200 randomly selected peaks with DNA sequences ±500 bp to the peak center) were evaluated, respectively. The putative direct/indirect binding sites were predicted in a previous publication [31] and were partially validated by wet-lab experiments. The mean and the standard deviation of the nTBA in the five sets are shown in Fig. 3b, which shows a clear difference of the nTBA effect to DNA between the direct and the indirect TF binding target sites. For low or median TF concentration (e.g., µ equals 0 or −10), there is a strong positive peak of nTBA at the center of the direct TF binding targets (both ER1 and SPIB). However, at the corresponding indirect TF binding targets (Fig. 3b), the effect of nTBA on DNA is similar to that of enhancers (Supplementary Fig. 2). For high TF concentrations (e.g., µ equals −13, −15, −18, −20), there is no effect of nTBA on DNA at both direct and indirect TF target sites (Fig. 3b). Thus, the effect of nTBA to DNA is clearly different between the direct TF-DNA interaction and the indirect TF-DNA interaction.

HOT regions are highly occupied TF target sites in the human genome, which were predicted by ENCODE project by considering observations of many TF ChIP-seq experiments in various human cell lines. To test the effect of nTBA on HOT regions, we randomly selected five sets of 200 HOT regions in the human genome. The mean and the standard deviation of the nTBA in five random selections are shown in Supplementary Fig. 2. Each selection contains ∼200 HOT regions with DNA sequences ±500 bp to the center of the HOT region. Here, only the nTBA estimated from low to median TF concentrations (e.g., µ equals 0 and −10) are considered because the additive effect is negligible at high TF concentration (e.g., Fig. 3a and b; Supplementary Figs. 1 and 2). These results reveal the effect of nTBA to DNA is stronger at HOT regions (Supplementary Fig. 2) than at the other regulatory regions (e.g., TSS, enhancer, and TF binding sites; Fig. 3a, and Supplementary Figs. 1 and 2). For example, there is a broader peak of the nTBA on HOT regions (e.g., ±200 bp to the HOT center with nTBA > 0.1; Supplementary Fig. 2) than that of TSS (e.g., from + 50 bp to −100 bp to the center of TSS with nTBA > 0.1; Fig. 3a and Supplementary Fig. 2). In other words, the effect of nTBA to DNA may contribute to gene regulation because it has a great influence on both the HOT regions and the gene regulatory regions (e.g. TF binding sites, TSS, and enhancer).

### 3.3 Non-specific TF binding affinity in human chromosomes

**Fig. 4:**
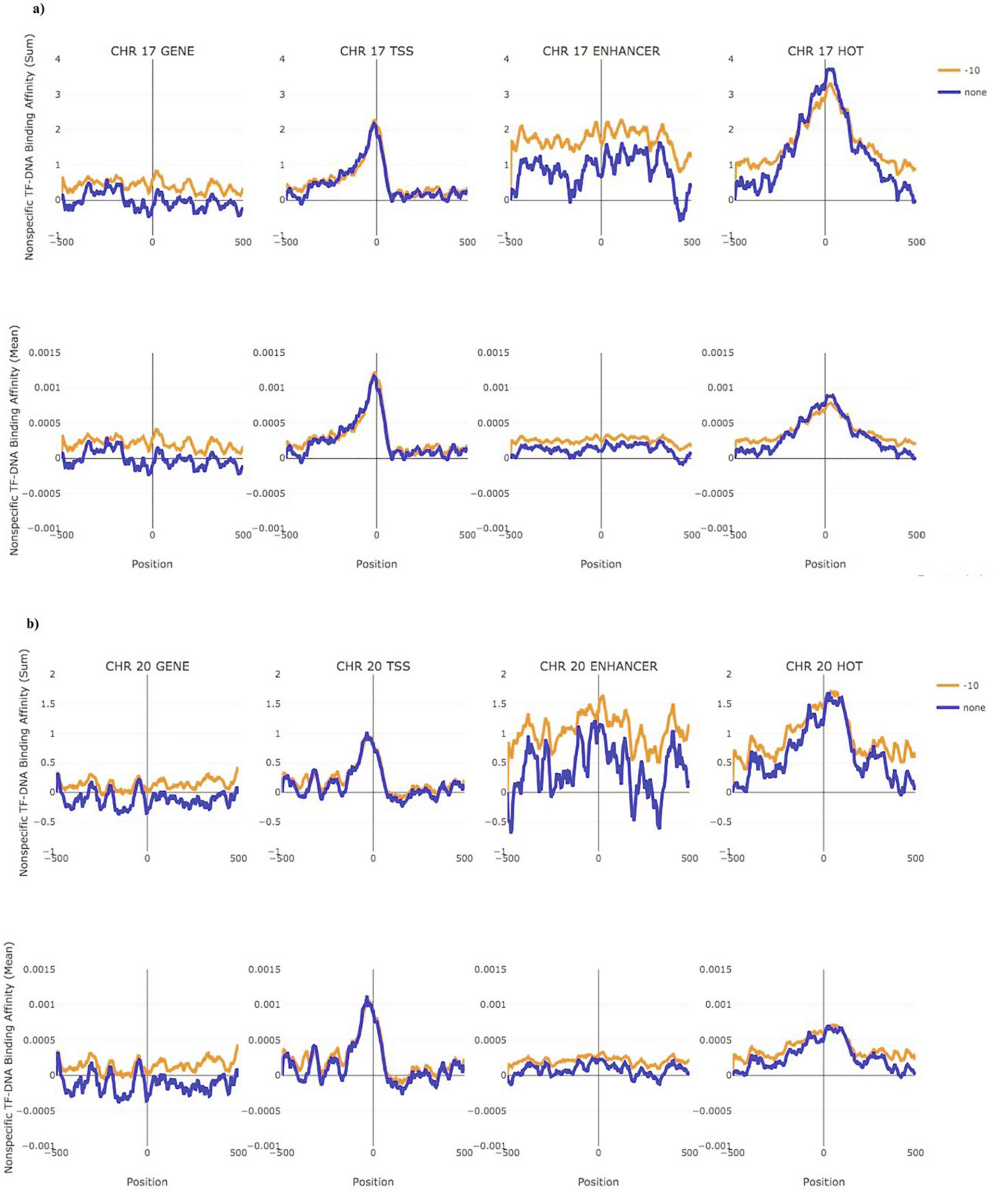
Distribution of nonspecific TF binding affinity at genes, transcription start sites (TSS), HOT regions, and enhancers in human chromosomes 17 and 20. For genes, TSS, HOT regions and the five types of tissue-specific human enhancers that are located on human chromosomes 17 and 20 (panel a and b respectively), we plotted the distribution of nonspecific TF binding affinity at ±500 bp centered on TSS or the center of gene/enhancer/HOT region, respectively. The first row (upper panel) for each chromosome presents the total sum of nonspecific binding affinities of each genomic feature (e.g., gene, TSS, enhancer or HOT region) while the second row (lower panel) represents the mean of nTBA, where 0 represents either the center of a gene, enhancer, HOT region or TSS. Predictions from different chemical potentials (or TF concentrations) are shown in different colors, respectively.

The total nTBA of ∼ 200 randomly selected sites, in various genome regions (e.g., gene, TF binding sites, TSS, enhancer, and HOT regions), indicates that there is an additive effect of nTBA to DNA. Such effect is very different between the gene regulatory region and the gene transcribed region (Fig. 3a and b; Supplementary Figs. 1 and 2). To further investigate this new DNA sequence feature, we computed nTBA for the whole genome (Fig. 4 and web Supplementary), at two TF concentrations (µ = 0 and −10), by using the proposed new biophysical model. Subsequently, the total nTBA to DNA in genes/TSS enhancers (e.g., thirty tissue-specific enhancers), and HOT regions on all human chromosomes are calculated. For each genomic feature (e.g., gene center, TSS, or enhancer), the pattern of the total nTBA from a chromosome (Fig. 4) is similar to that of the randomly selected ones (Fig. 3; Supplementary Figs. 1 and 2). Generally, the additive effect of nTBA to DNA is minimized in gene transcribed regions (e.g., oscillated around zero at gene center), has a narrow peak at TSS (e.g, >0.5 from −100 bp to 50 bp), fluctuates around a positive value at enhancer (e.g., around 1 and 0.5 for Chr17 and Chr20, respectively), and shows a strong and broad peak at HOT regions (e.g., >1 from ±200 bp to HOT center). These findings resemble the previous results from randomly selected sites, which support the hypothesis that there is an additive effect of nTBA to DNA in the regulatory regions such as a promoter, enhancer, and HOT regions.

Finally, the distribution of nTBA for the whole genome (except chromosome Y) at ±500 bp to the center of gene/TSS/enhancer/super-enhancer (SE)/hub-enhancer (HE)/HOT regions are illustrated in Supplementary Fig. 3a and b, respectively. In SFig. 3a, an additive effect of nTBA to DNA is observed in all regulatory regions. Here, SE is a cluster of enhancers which work together to control the gene regulation, and HE is a subclass of SE that overlaps with a genomic region having high chromatin interaction frequency [12]. Both SE and HE are computationally predicted from two cell lines (K562 and GM12878), the number of predicted SE/HE (<1000) is far less than the number of other genomic regions (e.g. enhancer > 40000 and HOT > 70000). Therefore, a comparison of mean nTBA between the SE/HE and the other genomic regions is suited. In SFig. 3b, the distribution of genome-wide mean nTBA in gene/TSS/enhancer/HOT regions resembles the corresponding chromosome-specific ones (Fig. 4 and web Supplementary). Though the pattern of genome-wide mean nTBA at SE and HE are similar (multiple peaks at ±500 to the centers), there is a clear difference between the SE/HE and the other regulatory regions (enhancer/TSS/HOT). Thus, our theoretically estimated nTBA on DNA sequences reflects the biological difference among various genomic regions.

### 3.4 A comparison between the non-specific TF binding affinity and the other genomic markers or epigenomic modifications in TSS and HOT regions

**Fig. 5:**
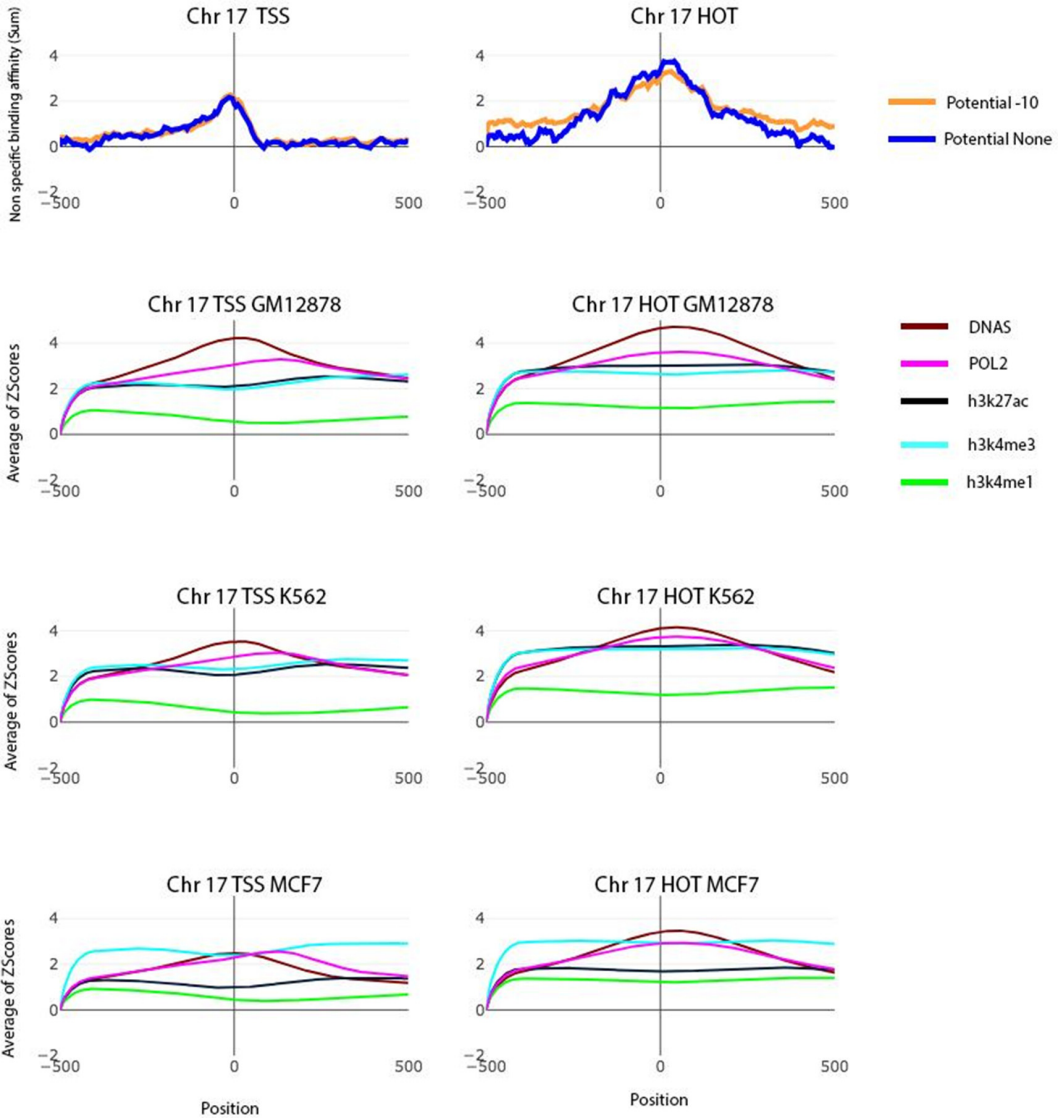

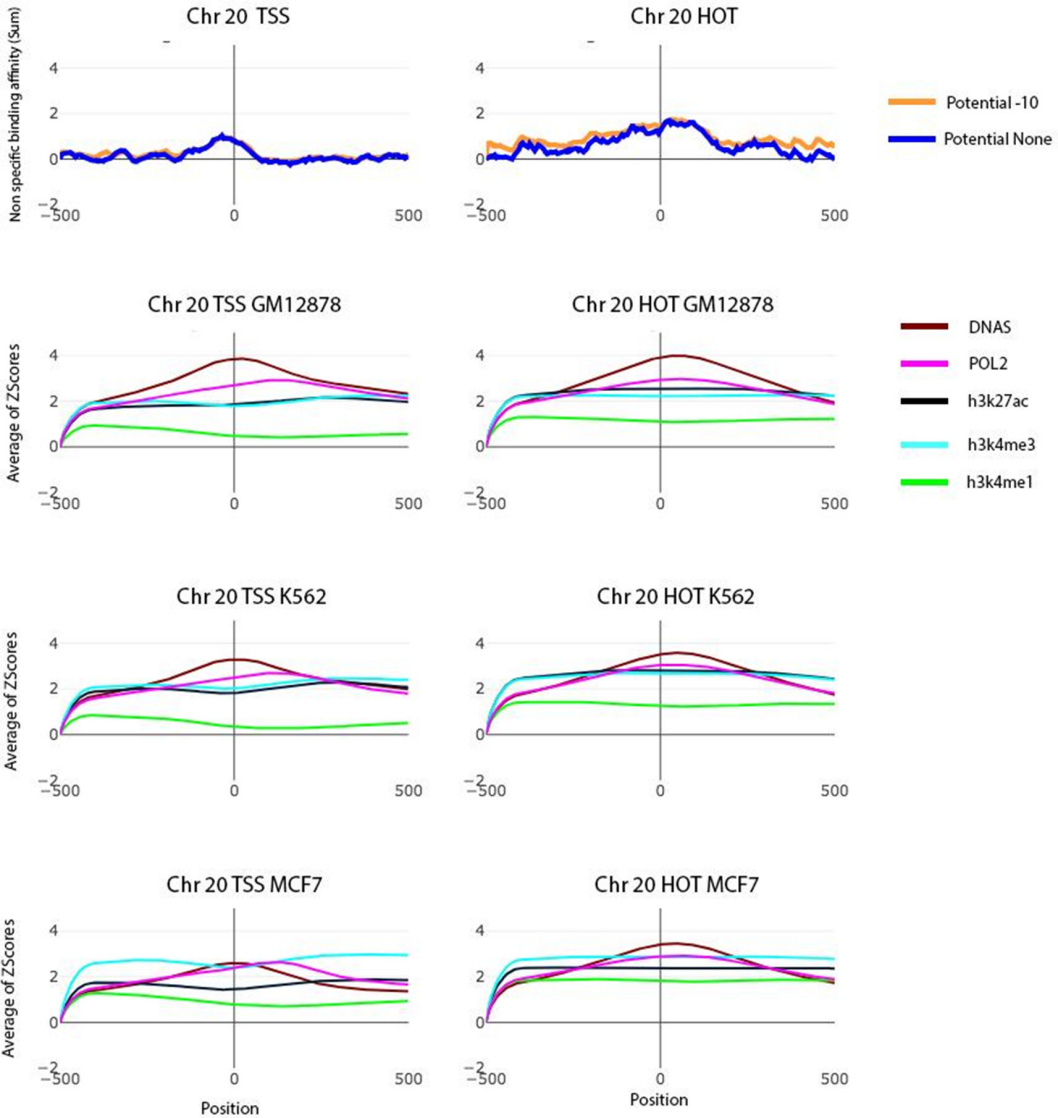
A comparison between the nonspecific TF-DNA binding affinity and the other genomic markers or epigenomic modifications at transcription start sites (TSS) and HOT regions of chr17 and chr20 For all TSSs and HOT regions located in human chromosome 17 and 20 (panel a and b respectively), the distributions of nonspecific TF-DNA binding affinity, histone modifications (H3k27ac, H3k4me1, and H3k4me3), Pol2 expression, and nucleosome occupancy (Pol2 and Dnase) in K562, GM12878, and MCF7 cell lines are illustrated, respectively. HOT regions are obtained from ENCODE predictions (e.g. P < 0.05 in any conditions). The average Z-scores of aforementioned modifications and the total nonspecific TF-DNA binding affinities are plotted in different colors, where zero represents either TSS or the center of HOT regions (e.g., ±500 bp from the center). H3K27ac and H3K4me1 are enhancer markers, H3K4me3 is promoter marker, Pol2 represents gene expression activity and Dnase indicates nucleosome occupancy in the genome.

The current study reveals the effect of nTBA to regulatory DNA sequences (e.g., TSS, enhancer, and HOT regions). Though the pattern of total nTBA in various regulatory regions is similar, the magnitude of the nTBA is always higher and broader at the HOT regions than that at the other regulatory regions (e.g., TSS; Fig. 4). Usually, there are more HOT than TSS regions in a chromosome (e.g., ∼4214 and ∼2404 HOT regions versus ∼1868 and ∼911 TSS ones in Chr17 and Chr20, respectively). Both the TSS and the HOT regions have a higher propensity to be bound by other TFs [64] because of the low nucleosome occupancy and presence of various TF binding sites at these genomic elements. Therefore, it is useful to investigate the relationship between the total nTBA and the other genomic markers or epigenomic modifications in both TSS and HOT regions. This may help us reveal a potential mechanism of HOT regions in the human genome. In Fig. 5, results of the total nTBA to TSS and HOT regions are shown for chromosome 17 and 20, where the additive effect of nTBA to DNA is much higher and broader in HOT regions than in TSS. At the same place, the distributions of the three histone modifications (e.g., H3K27ac, H3K4me1, and H3K4me3), Pol2 expression, and nucleosome occupancy Dnase hypersensitive sites) are investigated in basal K562, GM12878, and MCF7 cell lines, respectively.

In Fig. 5, an average of the Z-scores of each epigenomic modification in either TSS or HOT regions of chromosome 17 and 20 is displayed. It shows that the pattern of H3K4me3 is similar between the TSS and the HOT regions (cyan color line). However, for the other three modifications (e.g., pink, black, and green color lines represent Pol2 expression, H3K27ac, and H3K4me1, respectively), their activity profiles are higher in the HOT regions as compared to the TSS ones. Especially, the DNA sequences of the HOT regions are more accessible (brown color line represents DNase) than that of the TSS ones. To verify the findings from chromosome 17 and 20, the same analysis was repeated in all human chromosomes (e.g. web Supplementary figure for each chromosome) where the distribution of each epigenomic modification in both TSS and HOT regions (e.g. nTBA, H3K4me3, H3K27ac, H3K4m1, Pol2 expression, and nucleosome occupancy) seems to follow similar pattern. For example, both of the H3K4me3 and Pol2 expression levels are similar between the HOT and the TSS regions across the three cell lines. However, the total nTBA, the two distal regulatory marks (H3K27ac and H3K4me1), and the nucleosome free regions (DNAse hypersensitive sites) are stronger in the HOT regions (e.g., the average Z-scores of H3K4me1 > 1; Fig. 5) than in the TSS regions (e.g., Z-scores of H3K4me1 close to 0). Thus, the effect of nTBA in regulatory DNA sequences may participate in long distance gene regulation (or enhancer-promoter interaction) because there is a positive correlation between the total nTBA and the distal regulatory marks.

### 3.5 A comparison between the frequency of intra-chromosomal interactions and the frequency of highly enriched epigenomic modifications in genomic window bins

**Fig. 6:**
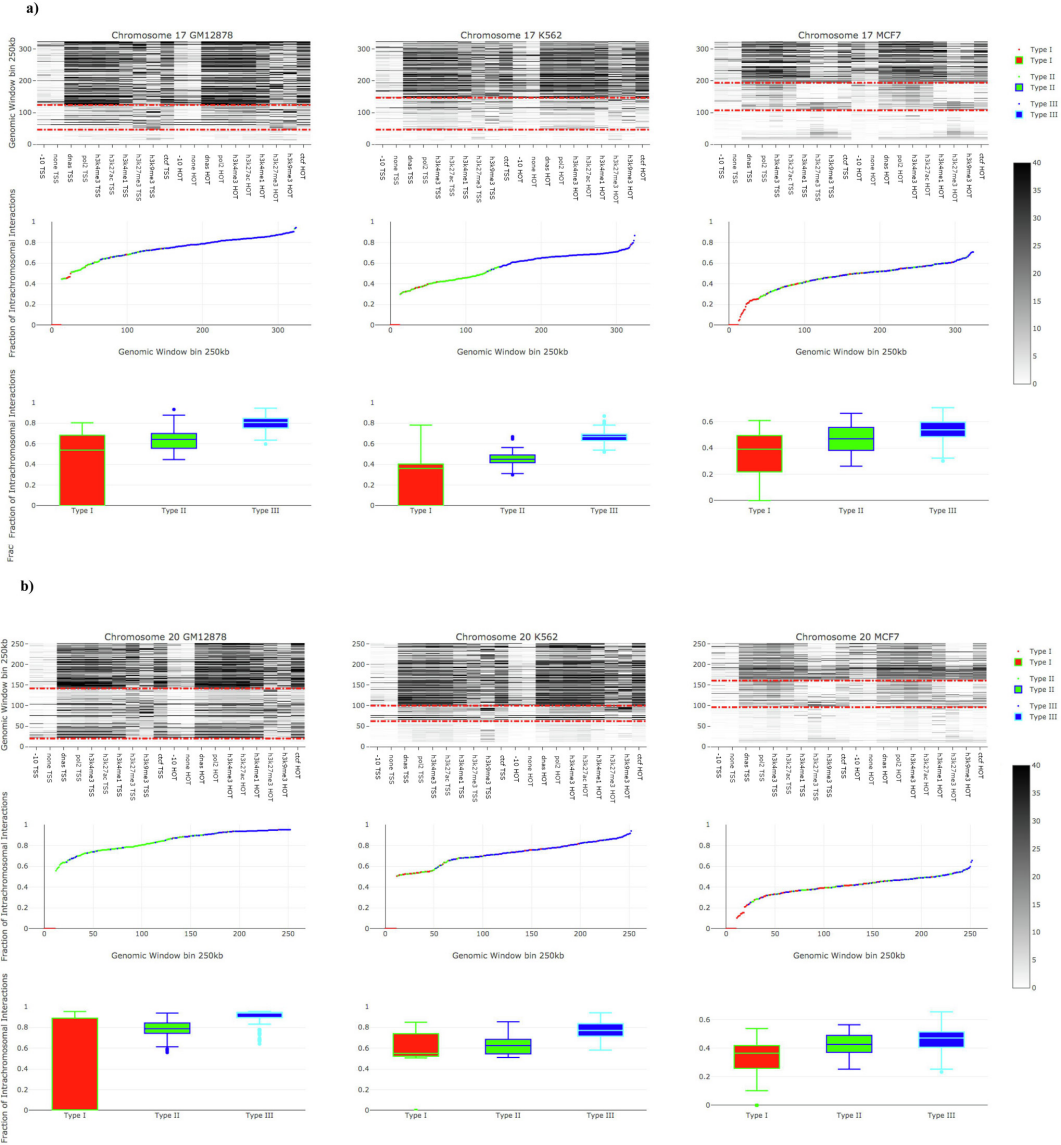
A comparison between the frequency of intra-chromosomal interactions and the frequency of highly enriched epigenomic modifications in three cell lines. In three cell lines, both the frequency of intra-chromosomal interactions (Z-score >0) and the frequency of highly enriched (Z-values ≥3) epigenomic modifications in genomic window bins (250 kb resolution) are shown for human chromosomes 17 and 20 in panel a and b, respectively. In the heat map, the three clusters are obtained by applying our clustering methods on the frequencies of highly enriched epigenomic modifications in window bins, where the clusters 1 (red), 2 (green), and 3 (blue) represent types I, II, and III genomic window bins, respectively, in intra-chromosomal interactions which drawn in box and scatter plot. Low, medium and high chromosomal interactions were classified as genomic window bins type I, II and III, respectively. (For interpretation of the references to color in this figure legend, the reader is referred to the web version of this article.)

An integrative data analysis in three cell lines (e.g., K562, GM12878, and MCF7) for all human chromosomes was performed to further investigate the role of nTBA in long distance gene regulation, by combining our data on nTBA to DNA with both the intra-chromosomal interactions (e.g., Hi-C experiment) and the other genomic markers or epigenomic modifications (e.g., H3K27ac, H3K4me1, H3K4me3, H3K27me3, H3K9me3, CTCF binding, Pol2 expression and DNase – nucleosome occupancy). Here, both the frequency of intra-chromosomal interactions and the frequency of highly enriched epigenomic modifications (e.g., Z-values ≥3) were computed in every genomic window bin (e.g., with resolution 250 Kb) for all human chromosomes in three cell lines (web Supplementary and SFigure 4). The frequency heat-maps of highly enriched epigenomic modifications in pair-wise intra-chromosomal interactions are shown in Fig. 6 for chromosome 17 and 20 only. Frequency heat-maps for the other chromosomes are presented in the Supplementary website.

By applying our modified K-means clustering algorithm on the frequencies of highly enriched epigenomic modifications in each chromosome, we identified three types of genomic window bins. The type I genomic window bins show no enrichment of active histone modifications in TSS regions (e.g., H3K4me1, H3K4me3, and H3K27ac) and very low frequencies of other highly enriched genomic marks such as CTCF binding sites, Pol2 expression and open nucleosomes. Furthermore, nTBA to HOT regions is not enriched in the type I genomic window bins. Type II genomic window bins show a low frequency of nTBA in HOT regions but a moderate frequency of highly enriched histone modifications (e.g., active marks - H3K4me1 and H3K27ac; repressive marks - H3K27me3 and H3K9me3), CTCF binding sites, Pol2 expression and open nucleosome regions in TSS. Type III genomic window bins show moderate frequencies of highly enriched nTBA and repressive histone modifications (H3K27me3 and H3K9me3) in both HOT and TSS regions, but high frequencies of highly enriched active histone modifications (H3K4me1, H3K4me3 and H3K27ac), Pol2 expression, CTCF binding sites and open chromatin in TSS regions. Thus, the frequency of intra-chromosomal interactions correlates with the frequency of highly enriched epigenomic modifications in the three genomic window bins; low, medium, and high frequency of intra-chromosomal interactions for the type I, II, III genomic window bins, respectively (Fig. 6).

### 3.6 A comparison between the three types of genomic bins and the other segmentations in human genome

**Fig. 7:**
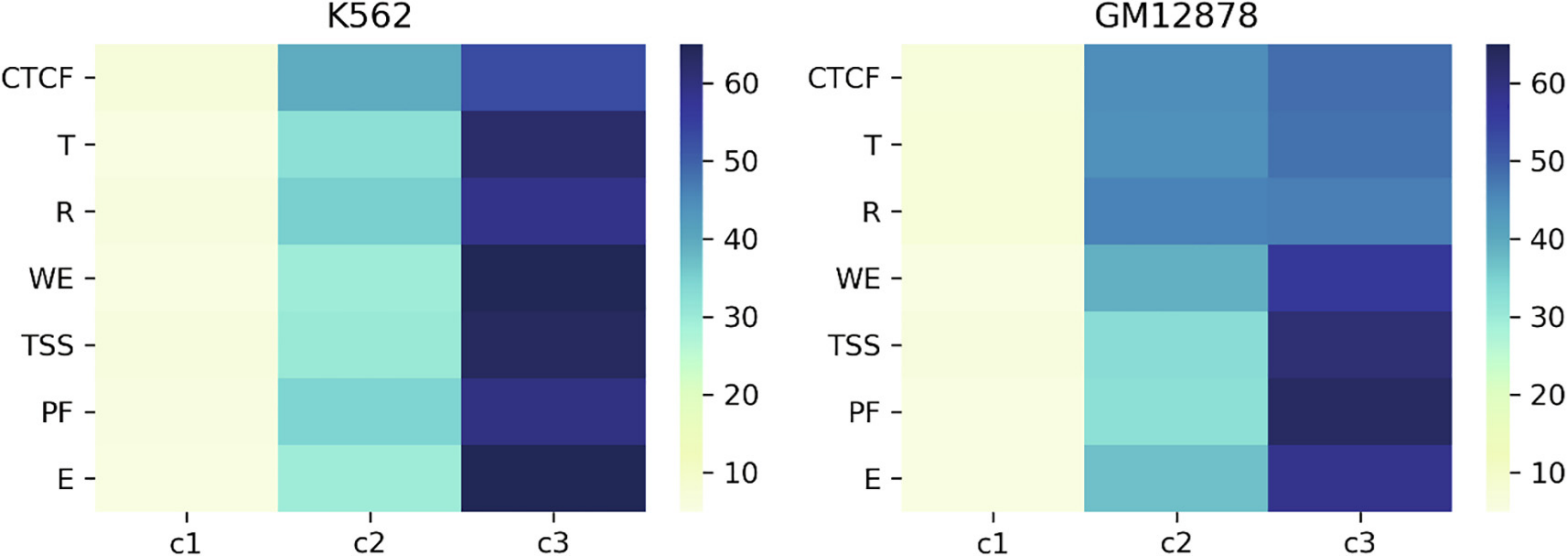
A comparison between the three types of genomic bins obtained using IGAP and the seven chromatin states predicted using machine-learning-based methods. The heat maps illustrate the percentage of overlap between one type of genomic bins classified by IGAP and a chromatin state predicted by both ChromHMM and Segway. C1, C2, and C3 represent type I, II, and III genomic bins obtained using IGAP in K562 and GM12878 cells, respectively. Seven chromatin states are: TSS – predicted promoter region including TSS; PF - predicted promoter flanking region; E – predicted enhancer; WE – predicted weak enhancer or open chromatin cis regulatory element; CTCF – CTCF enriched element; T – predicted transcribed region; R – predicted repressed or low activity region. The color shade represents percentage of chromatin state overlapping with one type of genomic bins. The darker cells the larger percentage.

The idea of genome segmentation is not new. There are several machine-learning-based methods (e.g., ChromHMM and Segway [38], [39]) available for segmentation of genome in functional regions based on chromatin features (e.g., histone modification and nucleosome occupancy). An earlier publication combined the human genome segmentations produced by ChromHMM and Segway software into a consensus. In the combined segmentations, seven chromatin states were used to segment human genome: TSS – predicted promoter region including TSS; PF – predicted promoter flanking region; E – predicted enhancer; WE – predicted weak enhancer or open chromatin cis regulatory element; CTCF – CTCF enriched element; T – predicted transcribed region; R – predicted repressed or low activity region. It is very interesting to compare the three types of genomic bins obtained by IGAP with the seven chromatin states produced using those machine-learning-based methods. Thus, the seven chromatin states in K562 and GM12878 cells are obtained from a previous publication [40] and are intersected with the three types of genomic bins in K562 and GM12878 cells, respectively, obtained using IGAP. First, the number of regions overlapping with each type of genomic bins is counted, then a percentage of each chromatin state that overlaps with each type of genomic bins is calculated. In Fig. 7, the percentages of chromatin states that are overlapping with the three types of genomic bins in K562 and GM12878 cells are illustrated in two heat maps, respectively. It suggests that the seven chromatin states have very low (<8%), marginal (∼30% to ∼40%), and high (∼50% to ∼65%) overlap with the type I, II, and III genomic bin, respectively.

Additionally, the distribution of nonspecific TF binding affinity at seven chromatin states obtained using machine-learning-based methods is also investigated in K562 and GM12878 cells respectively. For each predicted chromatin state, the mean and the standard deviation of the average nTBA from ten times randomly selected regions (e.g., ∼1000 in each, the mean nTBA at ±500 bp to the center of regions) in the whole genome are shown in supplementary Fig. 5. The mean nTBA profiles around the center of regions are quite different among various chromatin states. For example, there are often positive nTBA profiles in the regulatory regions (e.g. E, WE, PF, TSS, and CTCF). Especially, at median chemical potentials or TF concentrations (e.g. µ = −10), there is a positive peak in the center of regions. However, for putative transcribed region (T) and repressed or low activity region (R), the nTBA profiles are frequently negative and oscillate towards zero in low and median chemical potentials (SFig. 5), respectively. These observations are in line with our previous findings shown in Fig. 3, Fig. 4. It indicates that nTBA profile can be integrated with other chromatin features (e.g., histone modification and nucleosome occupancy) for the classification of the different chromatin functional states by using machine-learning-based methods.

### 3.7 An examination of the three types of genomic window bins

**Fig. 8:**
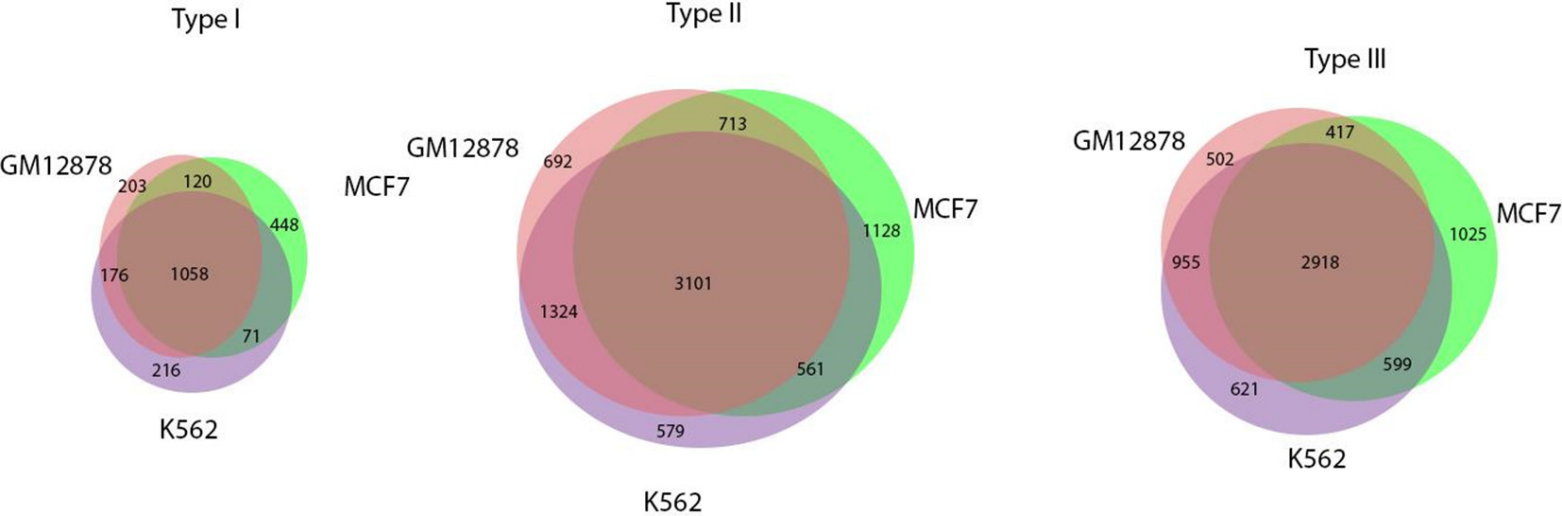
Overlapping intra-chromosomal interactions between three different cell lines for three types of genomic windows bins. For each type of genomic window bin, an intersection of windows in the three cell lines is shown.

Based on the frequency of intra-chromosomal interactions and the frequency of highly enriched epigenomic modifications (e.g., active/repressive histone modifications and nTBA in TSS/HOT regions; Fig. 5), the genomic window bins (e.g., 250 kb in resolution) were classified into three groups, respectively (Fig. 6). The results are consistent in three cell lines (MCF7, K562, and GM12878). Venn diagram analysis (Fig. 8) demonstrates a strong overlap of each type of genomic window bins in three cell lines; 1058 (62–69% of each cell line), 3101 (53–56% of each cell line) and 2918 (57–60% of each cell line) overlap for the type I, II, and III genomic window bins, respectively. These common bins are defined as the core type I/II/III genomic window bins. Since the frequencies of both the intra-chromosomal interactions and the highly enriched epigenomic modifications are low and marginal (Fig. 6) for the type I and the type II bins respectively, they may be associated with the “Inactive Genomic Zones” and the “Poised Genomic Zones”, respectively. For the type III genomic window bins, due to the high frequencies of both the intra-chromosomal interactions and the highly enriched epigenomic modifications, they may participate in the majority of intra-chromosomal interactions “Active Genomic Zones” and act similarly as the house-keeping genes in gene regulation.

**Fig. 9:**
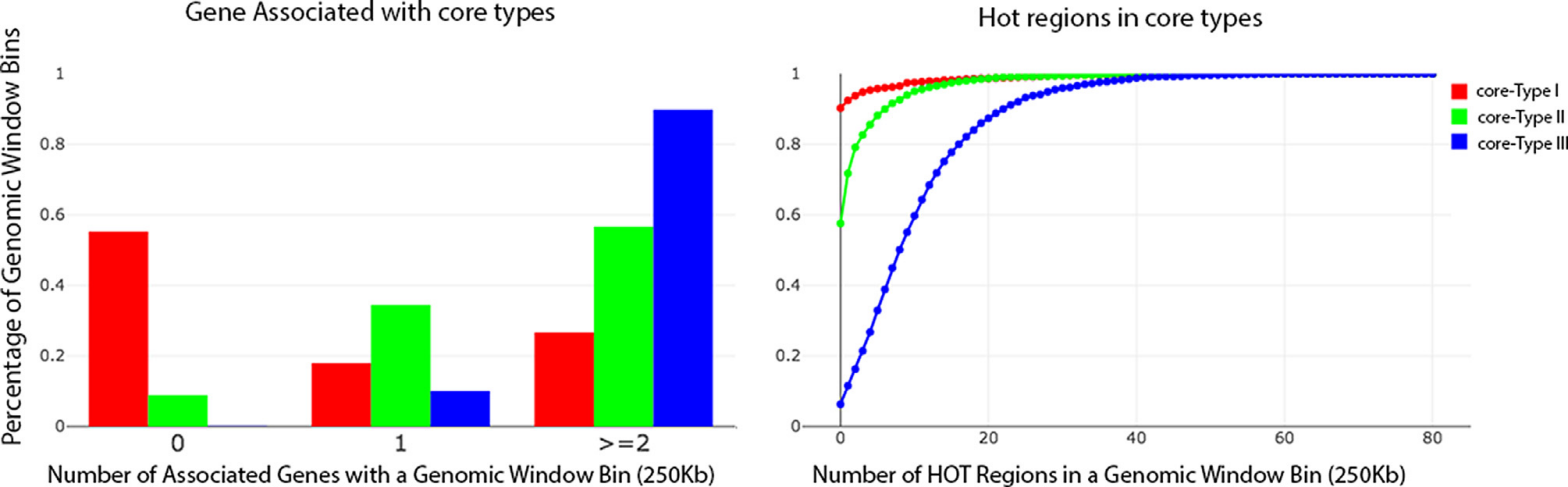
Number of genes and HOT regions associated with the three core types of genomic window bins. Here, the three core types of genomic window bins from all chromosomes are combined together, respectively. The numbers of genes and HOT regions associated with the core type I, II, and III regions are illustrated by bar plot (panel a) and empirical cumulative distribution function plot (panel b), respectively. The number of gene-core type associations is provided by the GREAT tool. HOT region-core type association is obtained from ENCODE predictions.

The core types I, II, and III genomic window bins (Fig. 8) exist in all three cell lines and are less affected by either experimental bias or other errors, which merits further examination. First, we used GREAT tool [65] to find gene-region associations with the core type I/II/III genomic window bins: about ∼55%, 9%, and 0% of the core types I, II, and III bins are not associated with any genes respectively, Supplementary files on website; however, ∼30%, ∼56%, and ∼90% of the core types I, II, and III bins are associated with two genes, respectively. In Fig. 9a, a bar plot of the number of associated genes with a genomic window bin is displayed, which indicates the core type I bin may be the least important region in intra-chromosomal interactions. That is because more than half (55%) of the core type I bins are not linked to any genes, but only ∼18% and 36% of them associated with one and two genes, respectively. On the contrary, all of the core type II/III bins are linked to genes and a majority (e.g., ∼56%/∼90%) of them are associated with two genes, which are more likely involved in functional intra-chromosomal interactions. Interestingly, 84% of the housekeeping genes overlap with the core type III bins substantiating a role of this core type in gene regulation, while only 11% and 5% of housekeeping genes overlap with core type II and I regions. Subsequently, an intersection between the three core types bins and the ENCODE HOT regions (71436) were computed; ∼10%, ∼43%, and 94% of the core type I, II, III bins are overlapping with the HOT region, respectively. In Fig. 9b, an empirical cumulative distribution of the number of HOT regions in every genomic window bin is illustrated. Notably, ∼1%, ∼9%, and ∼43% of the HOT regions are located in the core type I, II, and III bins respectively, which strongly suggests the intra-chromosomal interactions of the core type III bins may be very important in gene regulation because of the highest number of genes and HOT regions.

Finally, DAVID tools [60] are applied to analyze genes associated with the core type I, II and III genomic window bins, respectively. The enrichment of functional gene annotations (e.g., P-value <0.001) in the three core types of bins are listed in the Supplementary website. Gene ontology analysis of genes associated with the core type I bins (red color bars) showed enrichment of only three biological processes (e.g., cell/biological adhesion and neurological system processes) and no enrichment of cellular components and molecular functions (Supplementary Fig. 6). No annotation for KEGG pathways were found among core type I bins. Gene ontology analysis of core type II genomic window bins showed enrichment of 13 cellular components (majority related to membrane), 94 biological processes (i.e., regulation of nervous system development, appendage and ear morphogenesis, cell–cell signaling and adhesion), 16 molecular functions (i.e., sequence specific DNA binding, multiple channel activities, hormone binding etc) (Supplementary Fig. 6). KEGG pathway analysis of core type II associated genes showed only Neuroactive ligand-receptor interaction. Gene ontology analysis of genes associated with the core type III bins showed enrichment of 15 cellular components (i.e., cytosol, Golgi apparatus, cell projection, extra cellular matrix), 49 biological processes (i.e., cell motion and motility, regulation of processes like transcription, cell death and metabolism in GO biological process) and 11 molecular functions (i.e., nucleotide and nucleoside bindings, ATP binding, magnesium ion binding). KEGG pathway analysis of core type III bin genes showed six pathways associated with cancer (i.e., basal cell carcinoma, colorectal cancer) and Wnt signaling. Interestingly, more than 54% of biological processes related to core type II are development related suggesting that these genomic regions might become active in a spatiotemporal manner at specific developmental time points. The majority (34%) of biological processes related to core type III are regulation related (e.g. positive regulation of cellular biosynthetic process, regulation of transcription etc; Supplementary Fig. 6). Notably, more transcription factors binding sites are enriched in gene promoters associated with the core type III genomic window bins as compared to the other two core type bins. This result is supported by computational predictions from three tools (Supplementary Tables 2, 3, 4 and Supplementary website). It is evident that genes associated with the core type II/III bins are enriched in many molecular functions, biological processes, and cancer pathways. The results support our hypothesis that the type II and III bins (e.g., Poised/Active Genomic Zones) may participate in the functional intra-chromosomal interactions.

## 4 Discussions

In the current study, we have developed a pipeline for the integration of a wide variety of genomic data to reveal new insight about the regulatory landscape of the human genome. The Integrative Genome Analysis Pipeline (IGAP) combines a new DNA sequence feature nonspecific TF binding affinity (nTBA) with other genomic marks (e.g., histone modifications and intra-chromosomal interactions) to identify the genomic regions that are comparatively more robust in transcriptional activity. The theoretical foundation of nTBA calculation is similar to previous works [34], [55]. However, there are three major novelties in the current application: 1) the new biophysical model considers both very low (Maxwell-Boltzmann function) and general protein concentration (Femi-Dirac function) when computing nTBA; 2) the new model is implemented in the form of an efficient parallel computation algorithm that allows calculation of the nTBA in the entire human genome (over 3 billion bp), whereas the previous work [55] only tested ∼1600 yeast promoters with each sequence 100 bp long; 3) Integration of nTBA with other epigenomic modifications (IGAP pipeline) in the human genome, makes the first connection between the biophysical property of DNA sequences (nTBA) and the gene regulation in vivo, which not only contributes to understanding of the complicated 3-D diffusion process of TFs in search of true target sites but also provides hints about role of HOT regions in gene regulation.

Evaluation of the program on randomly selected sites from several human genome regions reveals interesting new insight about the impact of nTBA on DNA in these different regions. For instance, the effect of nTBA on DNA in gene centers is always very low, whereas the same effect in enhancer regions appears to be high in all cases. Both observations are biologically justified since TFs are not expected to bind in gene centers in most cases, whereas enhancer regions are crucial for TF binding. Moreover, TF binding sites are usually distributed uniformly and clustered multiple times across enhancers and hub/super-enhancers, respectively, which is in accordance with the uniform distribution and the multimodal distribution of nTBA in enhancer and hub/super-enhancers regions. Similarly, TF binding sites often reside upstream proximally to TSS, and the effect of nTBA increases as TSS approaches and decreases drastically downstream of TSS. The effect of nTBA in HOT regions is particularly interesting, where the cumulative nTBA is highest near the centers. This observation suggests a key role of nTBA in attracting a large number of TF to HOT regions in lack of canonical binding sites. Another observation is that nTBA to DNA is negligible at sites with high TF concentrations.

After revealing the different effects of nTBA to DNA in different genomic regions, the relationships between nTBA and the other genomic markers or epigenomic modifications were investigated in both TSS and HOT regions for all chromosomes, in three cell lines: 1) the additive effect of nTBA to DNA is higher in HOT regions than TSS, 2) DNA sequences are more accessible in HOT regions than TSS, 3) both H3K27ac and H3K4me1 levels are higher in HOT regions than TSS. It is known [3] that H3K4me3 is frequently enriched in active promoter regions, H3K27ac is an active enhancer marker which is found in both proximal and distal regions of TSS, and H3K4me1 is a histone marker linked to distal regulatory regions but is present at both active and poised enhancers. Additionally, Pol2 and nucleosome occupancy represent gene expression activity and accessibility of DNA sequences to other factors in the genome, respectively. Thus, the effect of nTBA to DNA in HOT regions is not only broader and stronger (Fig. 4) than TSS regions, but also associated with the enhancer like epigenomic modifications (Fig. 5) such as H3K27ac, H3K4me1 and nucleosome free regions. These findings point towards the potential role of nTBA in long distance gene regulation such as in functional intra-chromosomal interactions.

Subsequently, epigenomic modification enrichment tests in pair-wise intra-chromosomal interactions were performed based on the Hi-C experiments in the same three cell lines. In a pair of interacting genomic window bins, both the effect of nTBA and the other epigenomic modifications are considered in the TSS and HOT regions. The results suggest that there is a similarity between the enriched nTBA in HOT regions and the enriched epigenomic modifications in TSS with respect to intra-chromosomal interactions Supplementary Fig. 4. This is in coherence with the findings in Fig. 5, demonstrating a positive correlation between the nTBA to DNA and the enhancer markers (e.g., H3K27ac and H3K4me1). Thus, the effect of nTBA to regulatory DNA sequences may facilitate the recruitment of other TFs such as chromatin architecture proteins like CTCF to initiate correct histone modifications for controlling the long-distance gene regulation. It suggests that the functional intra-chromosomal interactions are not only related to epigenomic modifications (e.g., histone modifications, nucleosome occupancy, and CTCF binding [66]) in TSS/HOT regions but also are associated with the nTBA to DNA in TSS/HOT regions. The former one was reported previously by several other works [20], [21], [22], [67], [68], but the latter one – the contribution of nTBA to DNA in intra-chromosomal interaction – is a new discovery from the present study.

To further verify the findings, both the frequency of intra-chromosomal interactions and the frequency of highly enriched epigenomic modifications (e.g., Z-value of Rank-sum test ≥3) in genomic window bins (250 kb resolutions) were investigated. The classification of genomic window bins of chromosomes to three clusters, is based on the frequencies of 12 highly enriched features in pair-wise intra-chromosomal interactions: 1) four types of nTBA (e.g., in TSS/HOT regions estimated by either µ = 0 or µ = −10), and 2) eight types of epigenomic modifications. The latter category includes five active marks (e.g., nucleosome occupancy, RNA Pol2 expression, H3K4me3, H3K4me1, and H3K27ac), two repressive marks (H3K27me3 and H3K9me3) and a chromatin architecture protein CTCF that regulates 3D structure of chromatin. The result is consistent for all human chromosomes in three cell lines (Fig. 6 and Supplementary website). The frequencies of both the highly enriched 12 features and the intra-chromosomal interactions are low, marginal, and high in type I, II, and III genomic window bins, respectively. Especially, the frequency of the highly enriched nTBA to DNA is followed by the five highly enriched active marks and CTCF binding. Thus, low nTBA to DNA in TSS/HOT regions correlates with low active marks and intra-chromosomal interactions in these regions (e.g., the type I versus the type III genomic window bin in Fig. 6). Especially, in both K562 and GM12878 cell lines, there is a low, marginal, and high percentage of overlapping functional chromatin states (Fig. 7) with the type I, II, and III genomic window bins, respectively.

In Fig. 8 ∼66%, ∼55% and ∼59% of type I, II and III bins (respectively) of each cell line constitute the respective core types, which are further examined in detail. Generally, a low percentage of the core type I bins are associated with either gene (e.g., ∼26%; Fig. 9) or HOT regions (e.g., ∼10%; Fig. 9), but almost all of the core types II and III bins are associated with genes (e.g., ∼56% and 90% are linked to two genes in type II and III, respectively) and HOT regions (e.g., ∼43% and 92% in type II and III, respectively). Around half (∼43%) of the HOT regions in the human genome are located in the core type III regions, which is almost four times more than that in the other two core types (e.g., ∼1% and ∼9% for the core types I and II, respectively). Particularly, the frequencies of both the intra-chromosomal interactions and the highly enriched epigenomic modifications are low and marginal in type I and the type II bins, respectively. Considering the lack of genomic marks and intra-chromosomal interactions in core type I genomic window bins, we term them as “Inactive Genomic Zones”. On the other hand, core type II genomic window bins can be termed as “Poised Genomic Zones”, since they are equipped with genomic marks and features considerably higher than Inactive Genomic Zones, but they are more suitable for condition based activity. For the core type III genomic window bins, there are high frequencies of both the intra-chromosomal interactions and the highly enriched epigenomic modifications, and the core type III bins are associated with the highest number of genes (majority housekeeping) and HOT regions (Supplementary Figs. 7 and 8). These observations match earlier reports [67], [68] about chromosome interaction hotspots in human genomes, where the hotspots are associated with higher chromatin activity and transcription across cell types. The core type III bins may be the most active regions in intra-chromosomal interactions that contribute to long-distance gene regulation; here termed “Active Genomic Zones”. Functional annotation analysis of genes associated with the Inactive, Poised and Active Genomic Zones (core type I, II and III genomic window bins) was also performed (P-values <0.001, Supplementary Fig. 6). These results suggest that the Inactive Genomic Zones have a modest role in gene regulation, but both the Poised and Active Genomic Zones are active in many biological processes and signaling pathways. It implies that Active Genomic Zones may play a pivotal role in functional intra-chromosomal interactions and in controlling the long distance gene regulation.

**Fig. 10:**
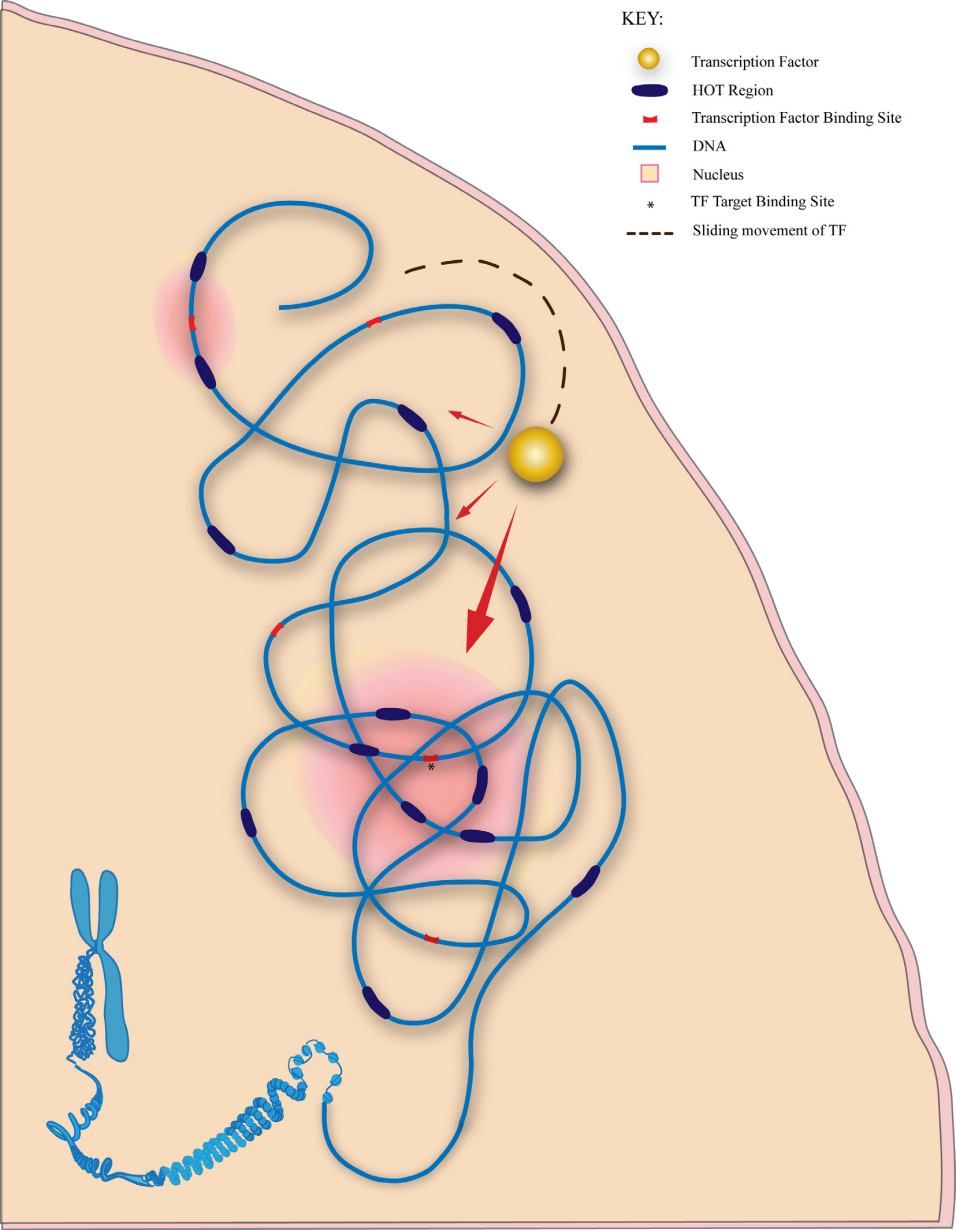
Illustration of HOT regions guiding a Transcription factor to its target binding site. HOT regions (purple) and binding sites (red) for TF are dispersed across DNA sequence. In a three-dimensional space, HOT regions come into proximity of the TF target binding site, generating higher TF binding potential in the region thus guiding the TF (yellow ball) to the actual target binding site (marked with *). Transcription factor which is sliding along DNA non-specifically (dashed line) in search of target site, faces a greater pull (large red arrow) towards the actual target binding site because of HOT regions gathered around it and has small attraction (small red arrows) for other binding sites. (For interpretation of the references to color in this figure legend, the reader is referred to the web version of this article.)

On the basis of all these findings, a new gene regulation model is proposed: 1) functional intra-chromosomal interactions reduce the physical distance between the HOT regions which increases the effect of nTBA to DNA; 2) the stronger effect of nTBA to DNA from the multiple HOT regions generates higher TF binding potential in the region, which facilitates recruitment of the TF towards the target binding site. This model is similar to a previous theory [69], suggesting that most of the TF enrichment within HOT regions may be caused by nonspecific DNA binding. Especially, the observation, from our integrated analysis of nTBA with chromatin modifications and chromosome interactions, supports the hypothesis in [69] that nonspecific TF-DNA interactions may guide TFs to find their true targets in the genome. Thus, our proposed new gene regulation model provides a new insight into the process used by TFs in search of their true target sites: for example, in a three-dimensional space of a nucleus, spatiotemporally specific intra-chromosomal interactions position the HOT regions in the vicinity of the true target sites and nTBA generated at these HOT regions is guiding TF binding to the true target sites as illustrated in Fig. 10. It is not unusual for HOT regions to mediate physical interactions between distant loci in the genome, considering similar behavior for several TF rich loci [70], [71]. HOT regions experience high Transcription Factor binding despite lack of clear sequence motifs [14]. Such non-motif binding of TFs at HOT regions can be explained by combination of two reasons. First, combinatorial collaboration of TFs using protein–protein interactions where only a few of the proteins are actually binding to the DNA [15], [72]. Unfortunately, only a handful such protein–protein interactions are experimentally verified[16], thus we kept our focus towards the second possibility which is existence of non-specific binding affinity that allows weaker binding of TFs to non-canonical motifs. In other words, given that HOT regions exhibit affinity for TFs despite the lack of TF binding sites, it is possible that HOT regions might play a role in recruiting the TFs to the target sites [15]. Therefore, the effect of nTBA to DNA may not only contribute to the functional intra-chromosomal interactions (the long distance gene regulation), but also can facilitate TF’s search for its true recognition sites on chromosomes.

## 5 Conclusions

This study proposes an Integrative Genome Analysis Pipeline (IGAP) based on a new biophysical model for estimating nTBA to DNA. Applying this biophysical model on human genome sequences unveils that nTBA to DNA has important regulatory functions. The integration of nTBA with other epigenomic modifications using IGAP revealed a positive correlation between the nTBA and the enhancer histone markers in intra-chromosomal interactions. Furthermore, human genomes can be clustered into three groups, by considering only the frequencies of highly enriched epigenomic modifications and the effect of nTBA at intra-chromosomal interactions.

Consequently, a new model of gene regulation based on the effect of nTBA to DNA has proposed: functional chromosomal interactions reduce the physical distance between the HOT regions that result in high nTBA to DNA in the area, which in turn attract TFs to such regions with higher binding potential. This phenomenon assists the correct matching of TF with its true target sites, reducing the search space for TFs. This new model reveals insights about the three-dimensional diffusion process of TFs, chromosomal interaction hotspots in a genome and the theory of transcription factories [73] that controls long distance gene regulation. Hence, IGAP provides a new and powerful tool to explore the complexity of genome regulation by employing features like nTBA and integrating multiple types of genomic data in a computational pipeline.

## Author contributions

ASN implemented the IGAP pipeline in Python and performed data analysis. AF provided data analysis tools, performed data analysis, and participated in writing manuscript. MB validated the study. JW conceived the project, designed IGAP pipeline, performed data analysis, contributed in developing Python package, drafted manuscript, and supervised the study. All authors read and approved the final manuscript.

## CRediT authorship contribution statement

**Alireza Sahaf Naeini:** Software, Validation, Formal analysis, Visualization. **Amna Farooq:** Visualization, Validation, Formal analysis, Writing - review & editing. **Magnar Bjørås:** Validation, Writing - review & editing. **Junbai Wang:** Conceptualization, Methodology, Software, Data curation, Writing - original draft, Visualization, Investigation, Formal analysis, Supervision, Validation, Project administration, Funding acquisition.

## Data Availability

The data, IGAP package, and figures supporting the findings of this study can be found at the Supplementary website (https://igap-pipeline.github.io/igap/).
