# Supplementary material for: IGAP-integrative genome analysis pipeline reveals new gene regulatory model associated with nonspecific TF-DNA binding affinity
Source: Comput Struct Biotechnol J. 2020 Jun 2;18:1270–86. doi: 10.1016/j.csbj.2020.05.024 (PMC7303559; doi:10.1016/j.csbj.2020.05.024)
Supplement: Supplementary data 1 [file mmc1.docx]

**IGAP - Integrative Genome Analysis Pipeline Reveals New Gene Regulatory Model Associated with Nonspecific TF-DNA binding affinity**

Alireza Sahaf Naeini^1,3^, Amna Farooq^1^, Magnar Bjørås^2,3^ and Junbai Wang^1*^

**Supplementary Methods**

**Obtain nonspecific TF binding affinity at randomly selected regions using IGAP**

We randomly selected a subset of regions from an input bed file (e.g., all TSS regions in a genome) to generate a representative nTBA profiles for the selected genomic region such as TSS. The random selection was repeated multiple times for computing the mean and standard deviation of nTBA profiles in the predefined region (e.g., +/-500 bp to the center of TSS). This function is available in the second step of IGAP, which can be run multiple times (e.g., 10 times per cell line, each time with 1000 randomly selected regions from an input bed file) with pre-computed nTBA values. Here, it first intersects nTBA result with the selected regions using bedtools. Then, nTBA are extracted from the selected regions (e.g., +/-500 bp to the center of TSS) and are further interpolated/smoothed using Python scipy interpolate library. Subsequently, the sum, the mean, and the standard deviation of nTBA profiles at randomly selected regions are calculated and illustrated in the supplementary Figures 1, 2, and 5. This process was done in each chemical potential, respectively.

**Supplementary Results**

## Non-Specific TF binding Affinity at selected Transcription Factor Binding Sites

To study the contribution of non-specific TF binding affinity (*nTBA*) to DNA in the human genome, ~200 called peaks from five human TF ChIP-seq experiments (e.g., CTCF, NRSF, STAT1, ER1, and SPIB) were randomly selected, respectively. For each TF, the distribution of the total *nTBA* at selected peaks is reported in Supplementary Figure 1 (e.g., +/-500bp to the called peak center), where the mean and the standard deviation of the total *nTBA* from multiple selections are shown. In the figure, the zero position represents the center of called peak, and the results estimated by different chemical potentials (or TF concentrations) are shown by different colors. Generally, there is a peak of the total *nTBA* in the center of ChIP-seq called peak (e.g., CTCF, NRSF, STAT1, ER1, and SPIB; Supplementary Figure 1). This indicates there may be an additive effect of *nTBA* (or total *nTBA*) to regulatory DNA sequences. Nevertheless, such an additive effect to DNA decreases, followed by an increase in TF concentration and almost disappears at high TF concentrations. For example, the total *nTBA* to DNA is almost zero (Supplementary Figure 1) when the chemical potential approaches -13, -15, 18, and -20, respectively. In other words, the additive effect of *nTBA* to DNA may only be valid from low to median TF concentrations (e.g. µ between 0 and -10). To prove the reproducibility of results, two sets of different sizes (~200 and 500 called peaks, in Supplementary figures 1b and c respectively) from the same five human TFs were randomly selected. These ChIP-Seq data sets are obtained from ENCODE STable 2, from two different cell lines for each TF. Here, the total *nTBA* was calculated for effective TF concentrations only (µ = 0 , -10). A general trend of the peak of the total nTBA can be observed around the center of ChIP-seq called peaks for nearly all TFs irrespective of the tissue specificity.

**Enriched epigenomic modifications in pair-wise intra-chromosomal interactions**

We hypothesized that the additive effect of *nTBA* to regulatory DNA sequences might contribute to long distance gene regulation and help TFs locate the true target sites. To verify this assumption, we performed an integrative data analysis in three cancer cell lines (e.g., K562, GM12878, and MCF7) for all human chromosomes, by combining the information of total *nTBA* to DNA with both the intra-chromosomal interactions (e.g., Hi-C experiment) and the other epigenomic modifications (e.g., H3K27ac, H3K4me1, H3K4me3, H3K27me3, H3K9me3, CTCF binding, Pol2 expression, and DNase - nucleosome occupancy). In Supplementary Figure 4, results of such analysis for chromosomes 17 and 20 are illustrated. In figures, the red-green and the yellow-blue heat-maps represent the Z-scores transformed Hi-C intra-chromosomal interaction contact matrix (e.g., the interaction is more and less frequent than the average of genome-wide interactions) and the Z-values of Rank-Sum enrichment tests (e.g., the positive and negative Z-values) of epigenomic modifications in pair-wise intra-chromosomal interactions, respectively. Here, the significance of epigenomic modification in a higher than genome-wide average Hi-C intra-chromosomal interaction (e.g., Z-scores>0) is compared to that of the expected background interactions (e.g., an average of epigenomic modifications in 100 randomly selected pair-wise intra-chromosome interactions).

In SFigure 4a, for three cancer cell lines, the enrichment tests of epigenomic modifications in chromosome 17 pair-wise intra-chromosomal interactions are displayed first. The results indicate there is a positive association between the intra-chromosomal interactions and the epigenomic modifications in TSS regions. For example, in higher than genome-wide average intra-chromosomal interactions (e.g. Z-scores>0; red color in SFigure 4), there are high enrichments of Pol2 expression, nucleosome open regions, CTCF binding sites, and histone modifications in the TSS regions. Especially for a pair-wise intra-chromosomal interaction, the enrichment of total *nTBA* in HOT regions is much higher than that in TSS ones. Also, for chromosome 17, the enrichment of total *nTBA* in HOT regions is similar to that of histone modifications in TSS regions (e.g., active markers - H3K4me3, H3K27ac, and H3K4me1; and repress markers - HeK27me3 and H3K9me3). In SFigure 4b, for the three cancer cells, results of epigenomic modification enrichment tests in chromosome 20 pair-wise intra-chromosomal interactions are illustrated. These results also suggest that the enrichment of total *nTBA* in HOT regions is similar to that of histone modifications in TSS. Thus, for intra-chromosomal interactions, there is a positive correlation between the total *nTBA* in HOT regions and the enhancer like histone modifications (e.g. H3K27ac and H3K4me1) in TSS. This is more evident when the *nTBA* is estimated from a reasonable TF chemical potential (e.g., µ=0 versus µ=-10 in SFigure 4a and 4b). This adds to our previous hypothesis that the additive effect of *nTBA* to DNA in HOT regions is as important as the histone modifications in TSS, which may contribute to functional long distance gene regulation for intra-chromosomal interactions. Supplementary Figures 4a and 4b reveal a correlation between intra-chromosomal interactions and epigenomic modifications in TSS regions. Interestingly, for a pair-wise intra-chromosomal interaction, the enrichment of nTBA is much higher in HOT regions than in TSS and similar to the enrichment of histone modifications in TSS regions. It implies that for intra-chromosomal interactions, there is a positive correlation between the nTBA in HOT regions and the enhancer like histone modifications in TSS. This finding supports our hypothesis that the effect of nTBA on DNA in HOT regions is as important as the histone modifications in TSS. Thus nTBA may contribute to functional long-distance gene regulation for intra-chromosomal interactions.

**A further examination of the three types of genomic window bins**

In order to further understand the connection between nTBA, genomic window bins, genes, HOT regions and intra-chromosomal interactions, all five were plotted against the ideogram of each chromosome for all three cell lines (SFigure 7). To emphasize the activity, house-keeping genes were plotted in addition to all RefSeq genes (SFigure 8). Regions with high nTBA (µ=-10) are always followed by the type II and type III window bins. Moreover, a high number of genes, especially house-keeping genes (Supplementary Figure 6) and HOT regions are also observed in the regions with high nTBA and vice versa. Similarly, intra-chromosomal interactions are also negligible in areas with low nTBA score, while they appear highly concentrated in areas with high nTBA. Interestingly, longer intra-chromosomal interactions (more than >10MB apart) are concentrated in Type III genomic window bins (high nTBA, genes, HOT regions) suggesting their role in long ranged intra-chromosomal interactions. Although intra-chromosomal interactions differ between the cell lines we observe a strong correlation between interactions, genomic window bins, nTBA, genes and HOT. This interesting correlation, in light of previous results, suggests a clear division of the human genome in three parts; Inactive Genomic Zones, comprising of type I genomic window bins where nTBA is the lowest and other genomic marks and features are also negligible in concentration; Poised Genomic Zones, consisting of type II genomic window bins characterized by considerable enrichment of nTBA, histone modifications, TF binding sites, genes, HOT regions and intra-chromosomal interactions, and associated with condition-specific activity; Active Genomic Zones, comprising of type III genomic window bins where high levels of nTBA is observed along with high enrichment of histone modifications, TF binding sites, HOT regions and intra-chromosomal interactions, and associated with especially house-keeping genes, and cancer related pathways.

In the Supplementary Figures 7 and 8, where high peaks of nTBA (red outer most rim) are always followed by the type II and III genomic window bins, and a high number of genes (green rim) and HOT regions (green rim). Interestingly, most of the long ranged intra-chromosomal interactions (green threads) are concentrated in the same regions as aforementioned. In contrast, the opposite trend is evident in the regions with low nTBA. In conclusion, a high number of housekeeping genes, HOT regions, regulation related GO:BP and cancer related pathways are strongly associated with Active Genomic Zones. It implies that Active Genomic Zones have high activity and regulatory roles, hence more prone to intra-chromosomal interaction. High frequencies of intra-chromosomal interactions in Active Genomic Zones indicate their role as chromosome interaction hotspots *^(^*[*^1^*](#_ENREF_1)*^,^* [*^2^*](#_ENREF_2)*^)^* and transcription factories in a genome *^(^*[*^3^*](#_ENREF_3)*^)^*. Hence Active Genomic Zones may play a pivotal role in functional intra-chromosomal interactions and in controlling the long distance gene regulation

**Supplementary Figures**

**SFigure 1. Distribution of nonspecific TF binding affinity at called TF peaks from ChIP-Seq experiments in human cells.**

For the Five Transcription Factors (CTCF, ER1, NRSF, SPIB, STAT1), we plotted the distribution of predicted nonspecific TF binding affinity calculated for +/-500 bp windows centered at the mean (indicated by 0 position) of called peaks in all figures a,b,c. The mean and the standard deviation of the total nonspecific binding affinities from five times randomly selected peaks (~200) for potential none, -10, -13, -15, -18, -20 are shown in the figure a. Figure b and c, represent the mean and the standard deviation of the total nonspecific binding affinities from five times randomly selected 200 and 500 peaks, respectively. Two conditions/ cell lines were chosen for each set of randomly selected peaks from ENCODE (e.g. GM12878 and K562 for CTCF in STable 2). Predictions from various chemical potentials (or TF concentrations) are colored by different colors, respectively.

**
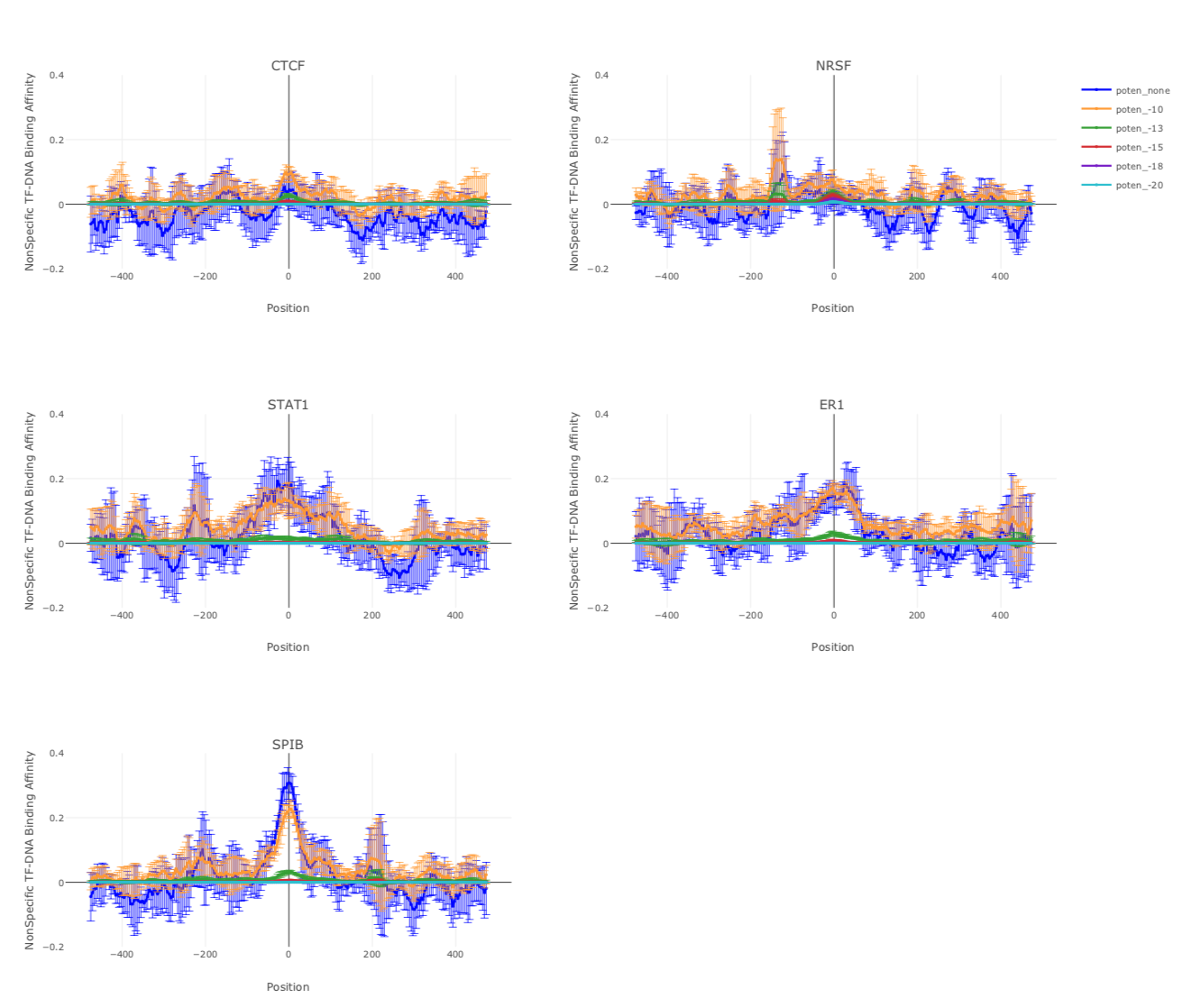
**

a)

**
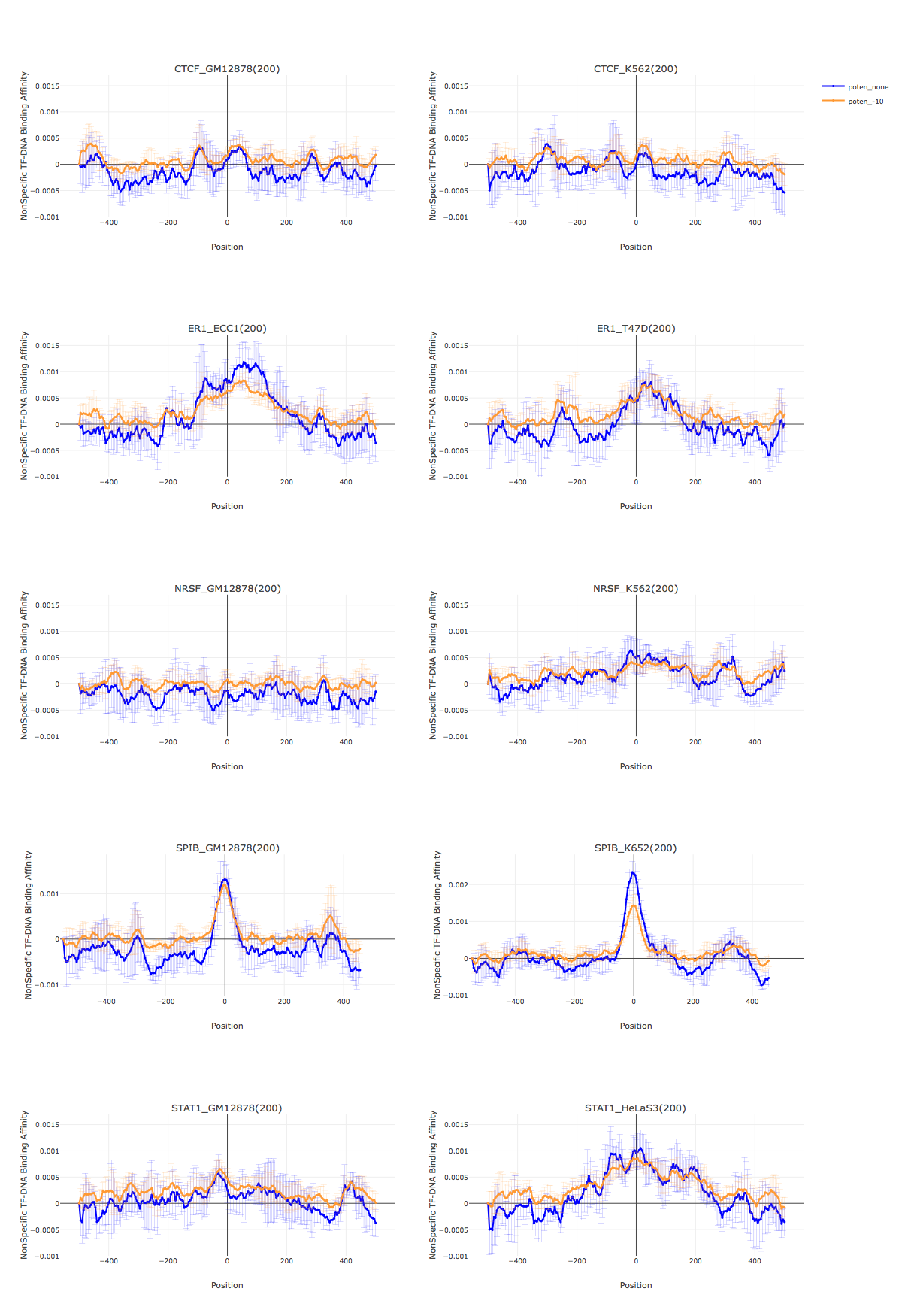
**

b)

**
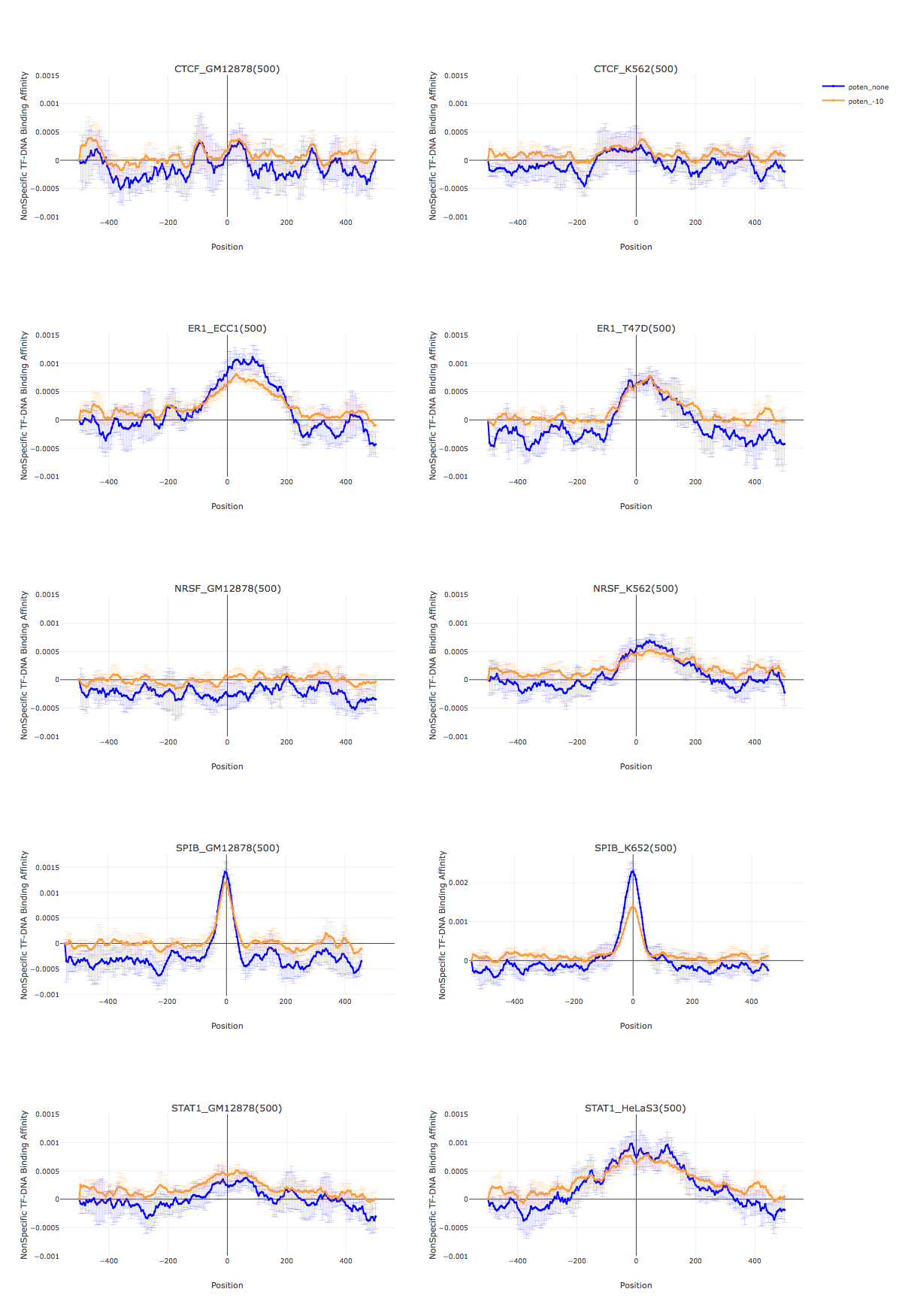
**

c)

**SFigure 2. Distribution of nonspecific TF binding affinity in human enhancers and HOT regions.**

Distribution of the nonspecific TF binding affinity at +/-500 bp to the center of HOT regions and enhancers for the whole human genome is represented bellow. The mean and the standard deviation of the total nonspecific binding affinities from five times randomly selected regions in the whole genome except chromosome Y (e.g., ~200 in each) are shown in the figure, where 0 represents the center of the region. Predictions from various chemical potentials (or TF concentrations) are represented by different colors, respectively.

**
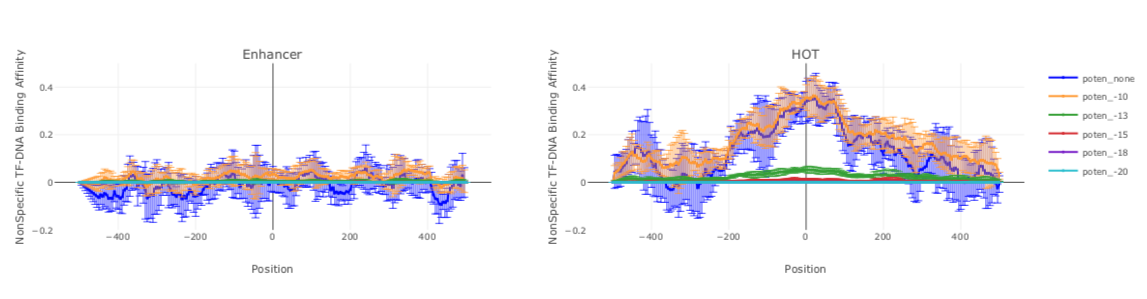
**

**SFigure 3. Distribution of genome-wide nonspecific TF binding affinity in human enhancer, gene, TSS, HOT, hub-enhancer, and super-enhancer regions.**

Here, distribution of genome-wide nonspecific TF binding affinity (nTBA) at +/-500 bp to the center of enhancer/gene/TSS/HOT regions/hub-enhancer/sup-enhancer for the human genome is illustrated. Figures a) and b) show the sum and the mean of the total nTBA in the whole genome except chromosome Y, respectively. In Figure, 0 represents the center of the region. Predictions from various chemical potentials (or TF concentrations) are represented by different colors, respectively.

a)


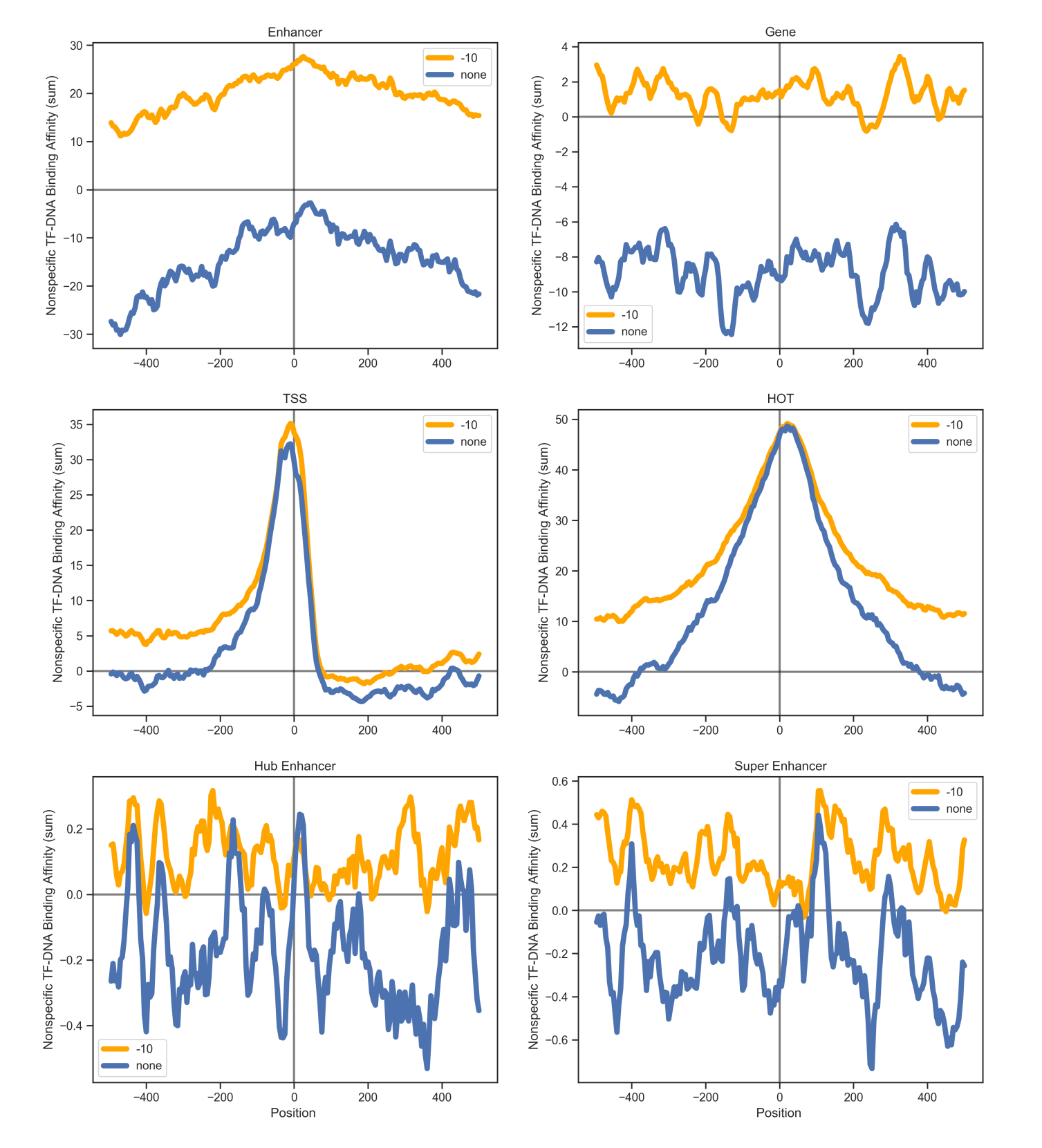


**
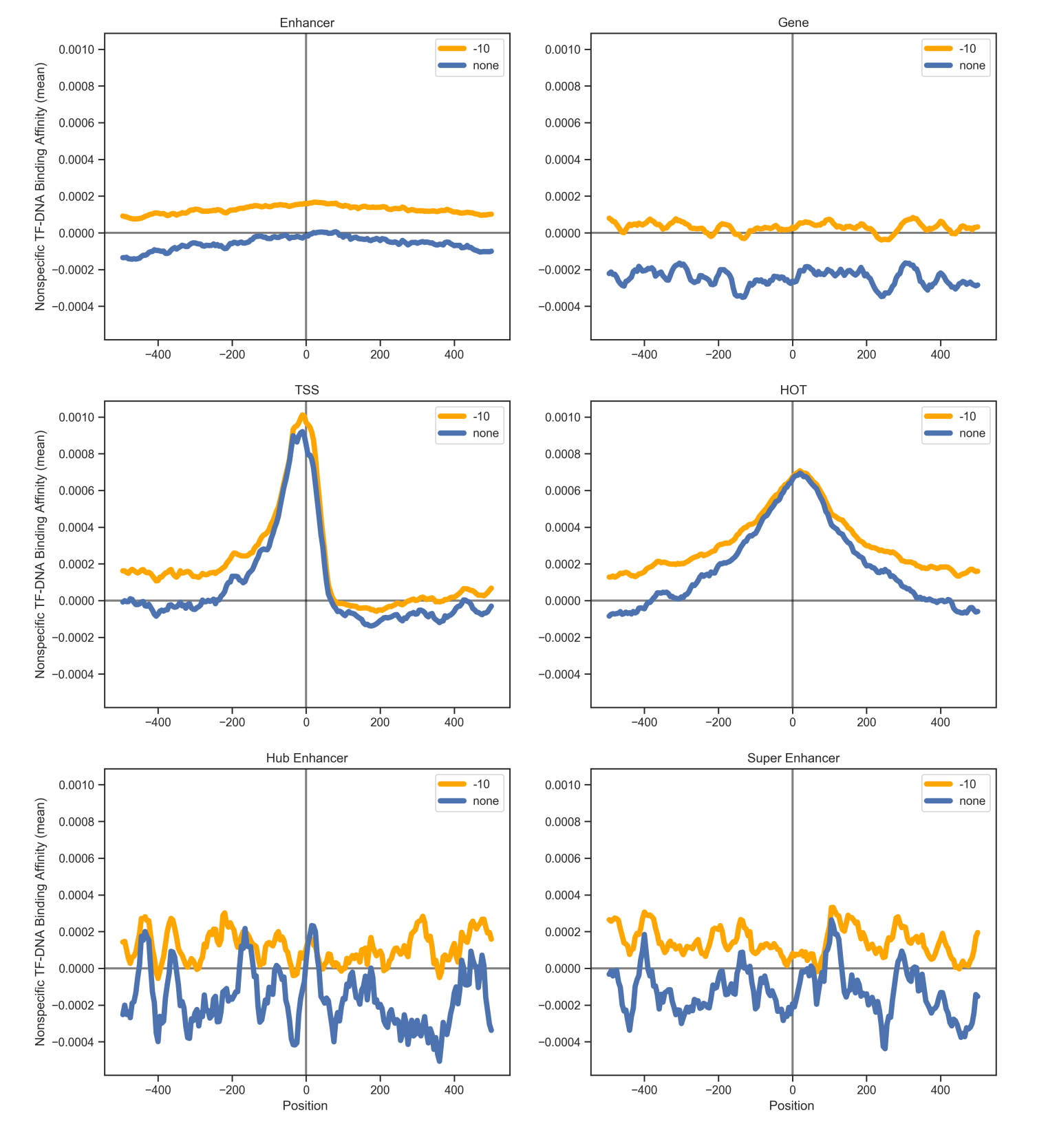
**

b)

**SFigure 4. Enrichment of epigenomic modifications in pair-wise intra-chromosomal interactions of chromosomes 17 and 20.**

For a pair-wise intra-chromosomal interaction, enrichments of nonspecific binding affinity, histone modifications (H3k27ac, H3k4me1, H3k4me3, H3K27me3, H3K9me3), CTCF binding, Pol2 expression, and nucleosome occupancy (Dnase) are tested based on Hi-C experiments of MCF7, K562, and GM12878 cell lines, respectively. Chromosome 17 is represented in panel a and chromosome 20 in panel b. Chromosomes are divided into equal sized bins (250Kb resolution) and experimental datasets are preprocessed, normalized, and converted to Zscores before further study. Here, a green-red color map represents a negative-positive Zscores from a Hi-C experiment: an interaction colored by red and green color means the interaction is more and less frequent than the average of genome-wide interactions, respectively. A yellow-blue color map represents a positive-negative Z-value from the rank-sum test. This is used to evaluate the enrichment of epigenomic modification in a pair-wise intra-chromosomal interaction (Zscore>0) versus that in background interactions such as the mean of 50 randomly selected intra-chromosomal interactions. In the figure, white and black color represents no interaction and no enrichment, respectively. Please note that we only consider epigenomic modifications around both TSSs and HOT regions (e.g., +/-500bp center) for a pair-wise intra-chromosomal interaction. If multiple TSSs or HOT regions located in the same window bin then an average of them will be used in the enrichment test. H3K27ac and H3K4me1 are enhancer markers, H3K4me3 is a promoter marker, H3K27me3 and H3K9me3 are genes repress markers, CTCF controls chromatin loops, Pol2 represents gene expression activity, and Dnase indicates nucleosome occupancy in the genome.

**
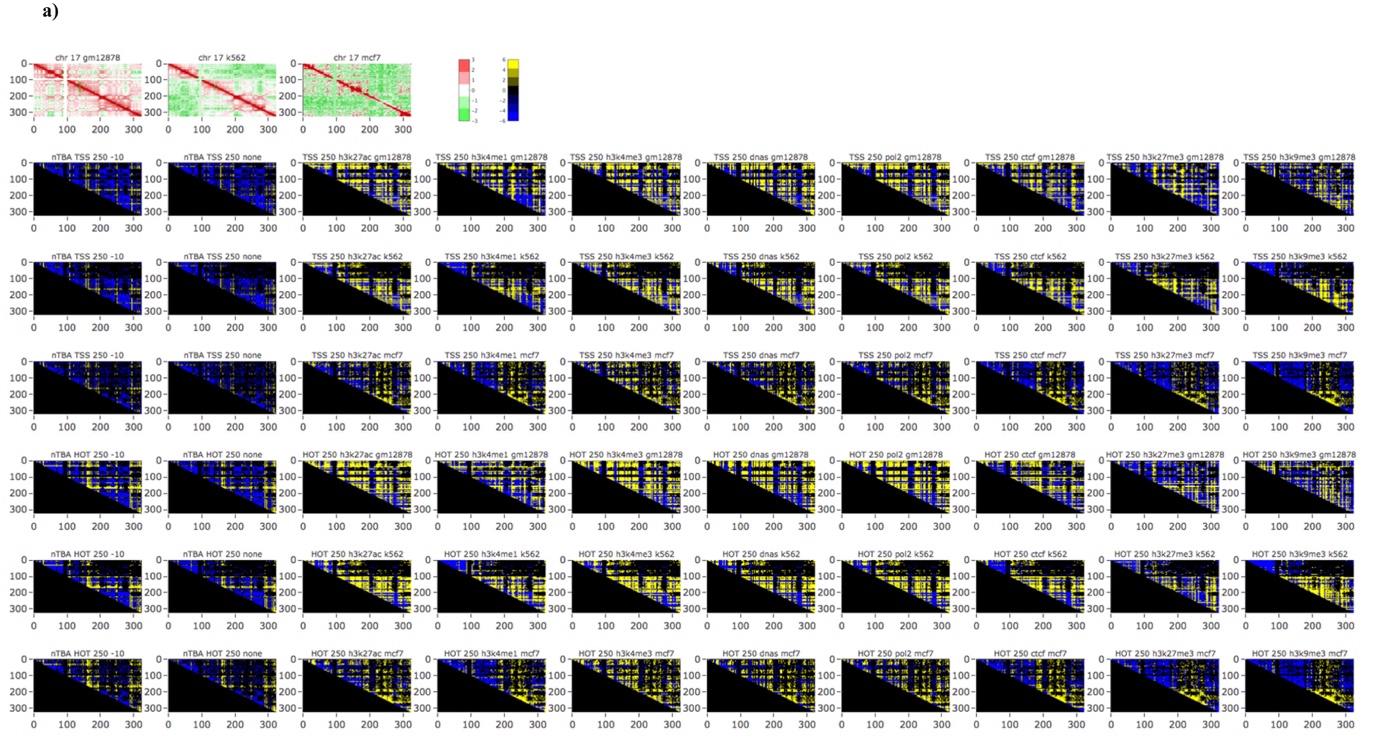

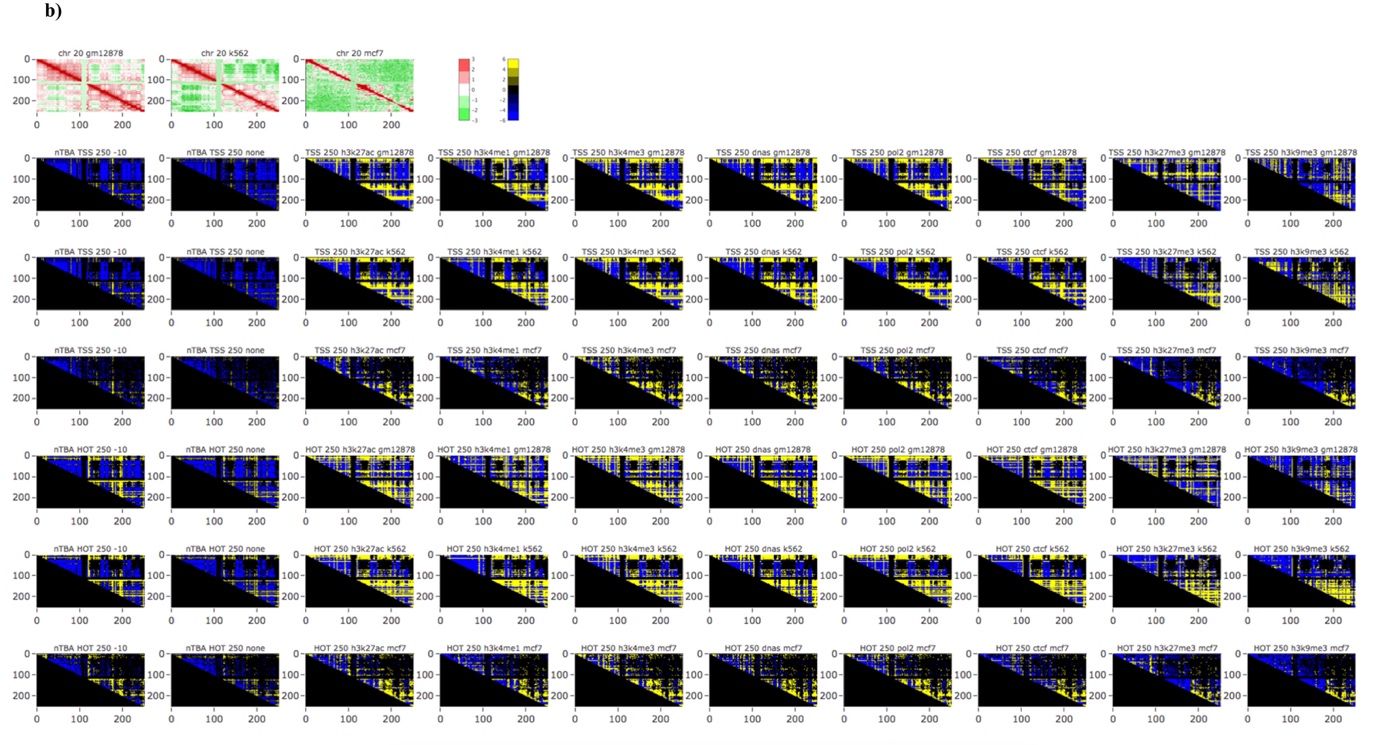
**

**SFigure 5. Distribution of nonspecific TF binding affinity in seven chromatin states produced by machine-learning-based methods**

Distribution of the nonspecific TF binding affinity at +/-500 bp to the center of seven chromatin states for the whole human genome is represented bellow. The mean and the standard deviation of the mean nonspecific binding affinities from ten times randomly selected regions in the whole genome except chromosome Y (e.g., ~1000 in each) are shown in the figure, where 0 represents the center of the region. Predictions from various chemical potentials (or TF concentrations) are represented by different colors, respectively. Here, the seven chromatin states were merged predictions from both ChromHMM and Segway software in K562 and GM12878 cells: E – predicted enhancer; WE – predicted weak enhancer or open chromatin cis regulatory element; PF - predicted promoter flanking region; TSS – predicted promoter region including TSS; T – predicted transcribed region; R – predicted repressed or low activity region; CTCF – CTCF enriched element.


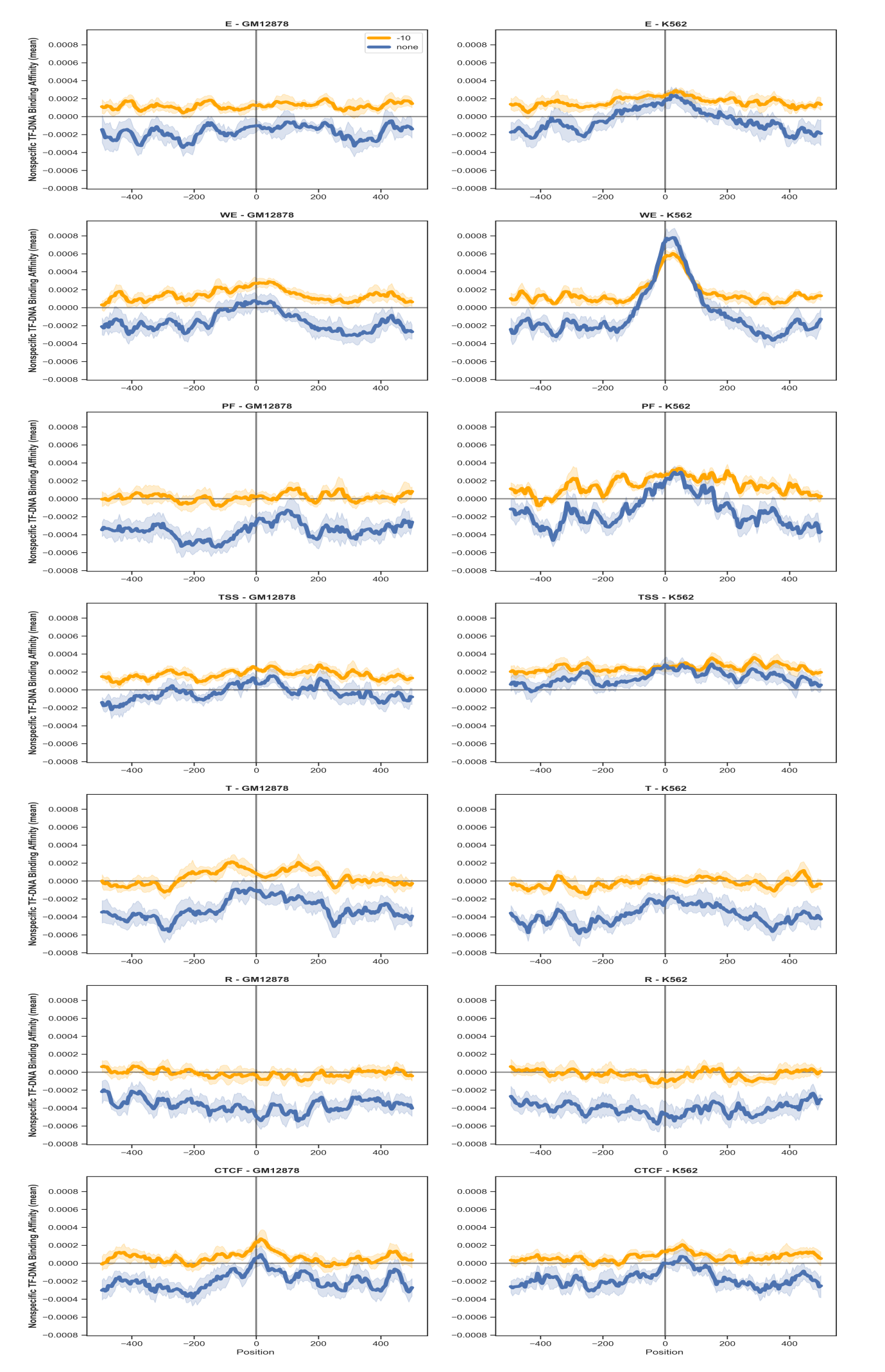


**SFigure 6. Functional annotation of genes associated with Core Inactive, Poised and Active Genomic Zones.**

log10(P)-value for functional annotation of genes associated with Core Inactive Genomic Zones are represented with red color bars, Core Poised Genomic Zones with Blue color bars and Core Active Genomic Zones with green color bars. Each graph represents all three annotation categories (Molecular Function in light blue, Cellular Compartment in pink and Biological Process in orange). X-axis represents log10(P)-Value, Y-axis represents the annotation term.


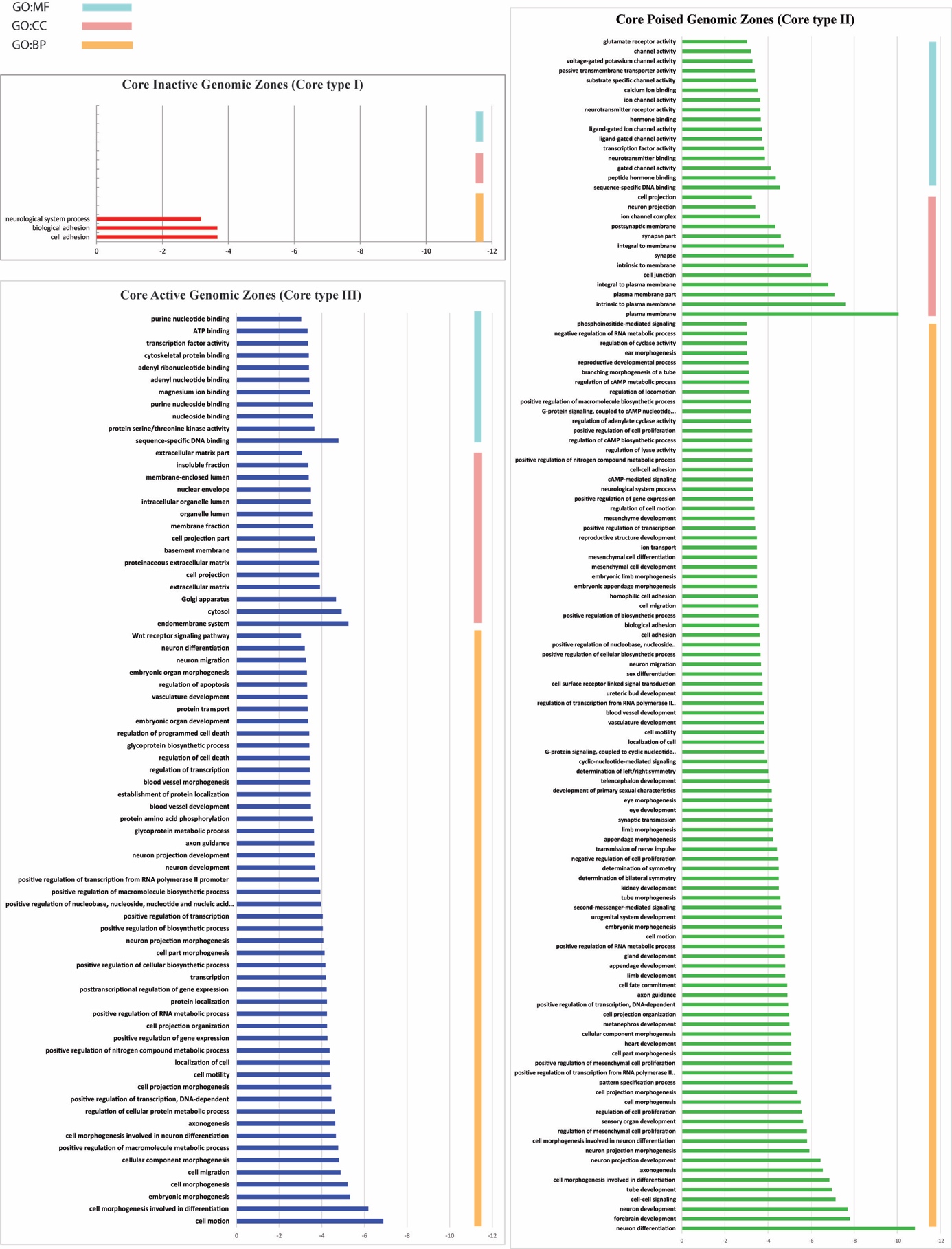


**SFigure 7. Comprehensive depiction of nTBA, genomic window bins and other genomic features in three cell lines for chromosome 17 and 20.**

The outer most white-grey banded circle is ideogram for chromosome 17. Second circle (red) represents nTBA for potential -10. Third circle (yellow) represents type I, II and III genomic window bins (red, green, blue tiles respectively). Fourth circle (green) represents number of genes. Fifth circle (orange) represents number of HOT regions. Green and Yellow threads in center represent intra-chromosomal HiC interactions with Z-score >= 1. All interactions between 1MB and 10MB are in yellow. Interactions spanning above 10MB are in green.

**
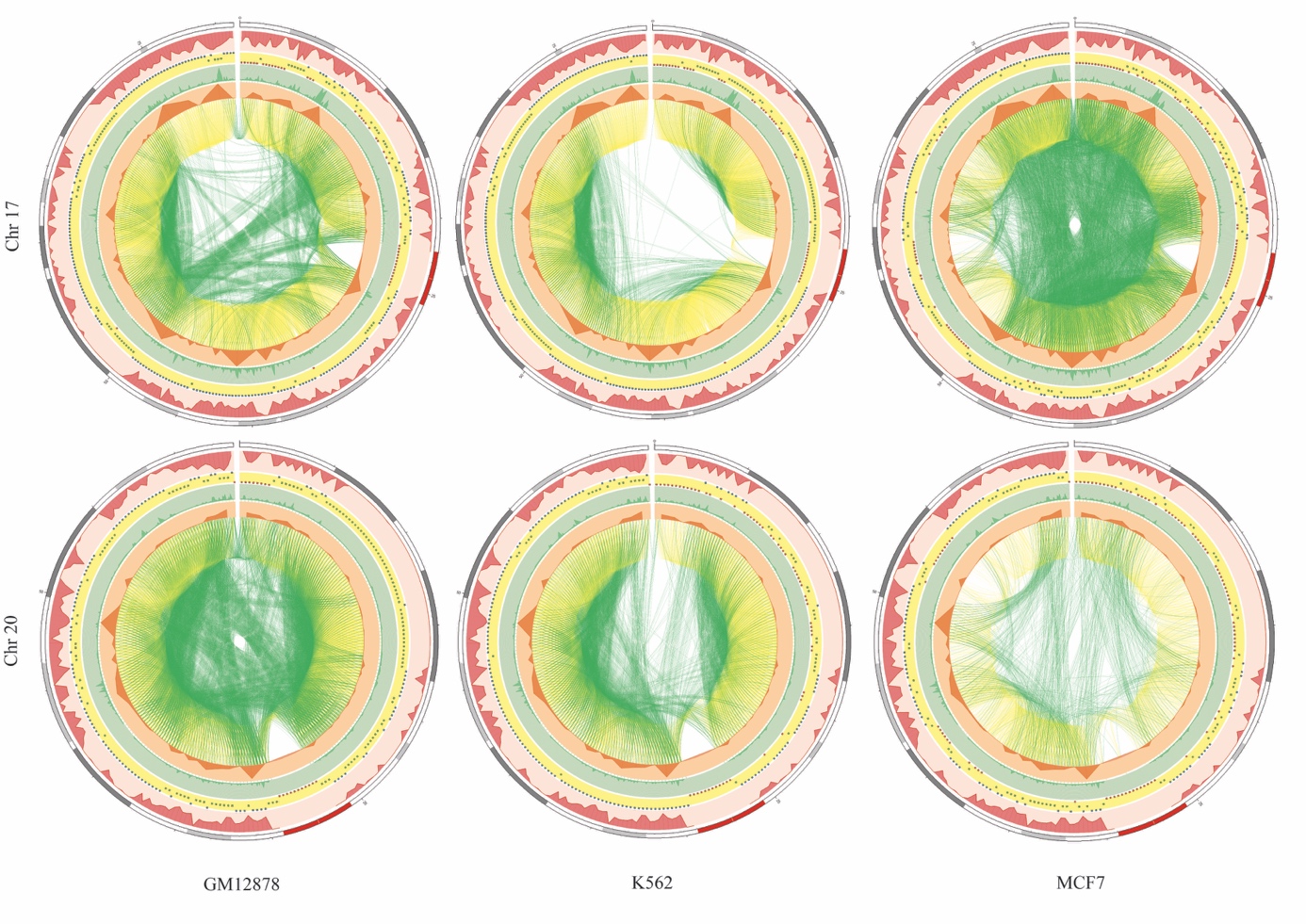
**

**SFigure 8. Comprehensive depiction of nTBA, genomic window bins and other genomic features in three cell lines for chromosome 17 and 20**

The outer most white-grey banded circle is ideogram of the chromosome. Second circle (red) represents nTBA for potential -10. Third circle (yellow) represents type I, II and III genomic window bins (red, green, blue tiles respectively). Fourth circle (green) represents the number of genes. Fifth circle (purple) focuses on house-keeping genes. Sixth circle (orange) represents number of HOT regions. Red and purple threads in center represent intra-chromosomal HiC interactions with Z-score >= 1.5. For the purpose of clarity, all interactions smaller than 2% of the size of the chromosome are not displayed. All interactions having distance larger than 10MB are in red. Interactions spanning smaller than 10MB are in purple. Figure 9 is built using the same parameters and data except for the circle for house-keeping genes is removed, intra-chromosomal HiC interactions with Z-score <= 1 and distance <1MB are not displayed for the purpose of clarity. Moreover, color code for HiC interactions is also different (yellow and green) for the purpose of clarity.


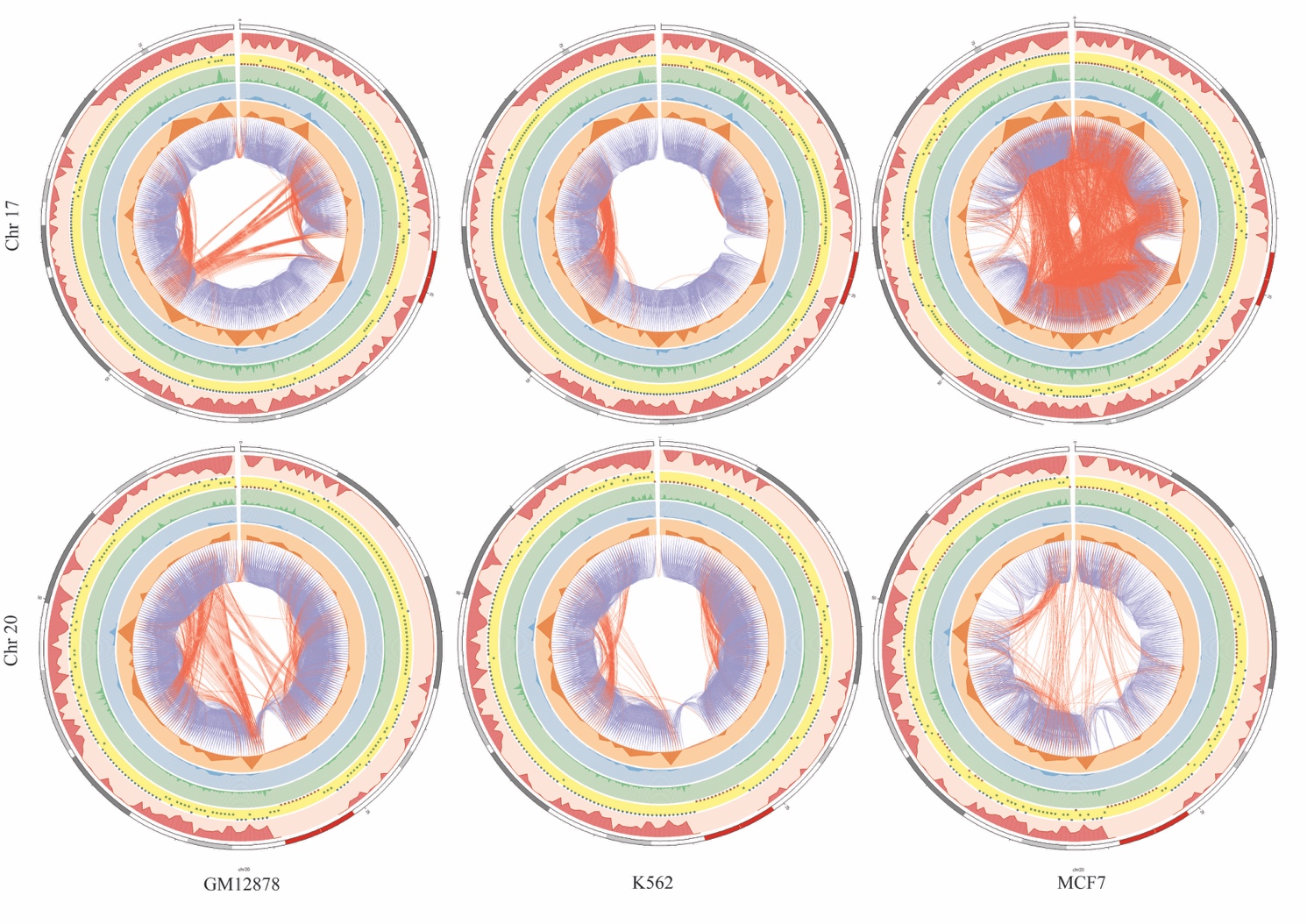


**Supplementary Website**

Supplementary website (https://igap-pipeline.github.io/igap/) contains data and figures for all chromosomes. Main figure 4, 5, 6 and Supplementary SFigure 4, 7 and 8 for rest of human chromosome (except Chromosome Y) are available on website. Genomic window bins for all three core types (Core Inactive, Poised and Active Genomic Zones) are available on website. Gene annotation data set for SFigure 4 is also available. Complete IGAP python package is also accessible for download on the website.

**Supplementary Tables**

**STable 1. Transcription Factor Binding ChIP-seq called peak sources from ENCODE.**

| TFs | Cell Line | GEO Accession number |
| --- | --- | --- |
| STAT1 | GM12878 | GSM935612 |
|  | HeLaS3 | GSM935360 |
| CTCF | GM12878 | GSM935611 |
|  | K562 | GSM935407 |
| NRSF | GM12878 | GSM1010744 |
|  | K562 | GSM803440 |
| SPIB/PU1 | GM12878 | GSM803531 |
|  | K562 | GSM803384 |
| ER1/ER alpha | ECC1 (Estradiol 10nM) | GSM803422 |
|  | T-47D (Estradiol 10nM) | GSM803539 |

**STable 2. DAVID - Transcription Factor Binding Site enrichment in gene promoters associated to respective core type genomic bins**

Enrichment analysis of TFBSs associated with core type genomic bins was done using DAVID tools. Binding site data is sourced from UCSC TFBS conserved track. Results shown are filtered for P-value <0.001. The results are purely computational and not all binding sites listed here are biologically functional binding sites.

| Type I | | Type II | | Type III | |
| --- | --- | --- | --- | --- | --- |
| TF | **P-value** | **TF** | **P-value** | **TF** | **P-value** |
| LHX3 | 1,31E-04 | LHX3 | 1,13E-199 | NKX61 | 7,80E-93 |
|  |  | NKX61 | 2,58E-162 | POU3F2 | 1,02E-92 |
|  |  | POU6F1 | 1,02E-161 | NKX3A | 4,62E-83 |
|  |  | CDC5 | 6,84E-161 | CDC5 | 1,72E-82 |
|  |  | S8 | 1,64E-160 | FOXJ2 | 2,73E-82 |
|  |  | TBP | 1,68E-149 | HNF1 | 3,17E-82 |
|  |  | NKX3A | 1,62E-145 | POU6F1 | 1,26E-81 |
|  |  | POU3F2 | 5,37E-144 | RSRFC4 | 5,78E-80 |
|  |  | CART1 | 1,35E-137 | CART1 | 7,79E-77 |
|  |  | FOXD3 | 5,74E-137 | FREAC7 | 1,33E-76 |
|  |  | FREAC7 | 1,17E-129 | MEF2 | 1,35E-76 |
|  |  | HFH3 | 3,32E-128 | HFH1 | 1,69E-74 |
|  |  | HFH1 | 9,58E-128 | CDP | 7,96E-73 |
|  |  | SOX5 | 1,41E-126 | SRY | 9,44E-73 |
|  |  | HNF3B | 2,40E-126 | LHX3 | 8,68E-72 |
|  |  | CDP | 2,93E-122 | NKX25 | 1,91E-70 |
|  |  | HNF1 | 7,04E-121 | S8 | 6,50E-70 |
|  |  | E4BP4 | 8,38E-120 | SOX5 | 1,50E-69 |
|  |  | CHX10 | 3,77E-115 | EVI1 | 9,92E-69 |
|  |  | FOXJ2 | 2,59E-111 | okt.01 | 3,68E-68 |
|  |  | RSRFC4 | 2,31E-110 | E4BP4 | 2,14E-67 |
|  |  | SRY | 1,10E-109 | HFH3 | 4,99E-67 |
|  |  | PBX1 | 3,39E-106 | FOXO4 | 8,50E-64 |
|  |  | NKX22 | 4,90E-105 | HNF3B | 1,59E-63 |
|  |  | FOXO3 | 2,71E-98 | OCT | 1,03E-62 |
|  |  | FREAC3 | 1,23E-96 | FOXD3 | 1,67E-62 |
|  |  | OCT | 1,21E-95 | TATA | 1,36E-61 |
|  |  | FOXO1 | 7,69E-95 | CHX10 | 3,87E-60 |
|  |  | FREAC2 | 6,25E-94 | FOXO1 | 1,30E-56 |
|  |  | HLF | 8,58E-92 | NKX22 | 3,01E-55 |
|  |  | BRN2 | 2,20E-88 | HLF | 4,35E-54 |
|  |  | TATA | 3,03E-85 | NFAT | 3,13E-53 |
|  |  | MEIS1BHOXA9 | 2,70E-84 | FREAC3 | 2,96E-52 |
|  |  | CEBPA | 3,14E-84 | PBX1 | 6,71E-50 |
|  |  | MEF2 | 1,38E-82 | TBP | 2,20E-49 |
|  |  | FREAC4 | 9,64E-81 | GATA | 8,37E-48 |
|  |  | NKX25 | 3,02E-80 | CEBPB | 2,56E-47 |
|  |  | FOXO4 | 5,56E-80 | IRF1 | 3,07E-45 |
|  |  | SOX9 | 4,86E-76 | BRN2 | 2,84E-43 |
|  |  | NFAT | 1,53E-72 | IRF2 | 9,16E-43 |
|  |  | STAT5B | 4,10E-71 | CEBP | 1,34E-42 |
|  |  | GATA | 9,28E-66 | SOX9 | 2,54E-41 |
|  |  | IRF1 | 2,51E-64 | FREAC4 | 1,45E-40 |
|  |  | EVI1 | 1,07E-57 | CREBP1 | 1,27E-39 |
|  |  | RORA2 | 9,55E-57 | MEIS1BHOXA9 | 1,70E-39 |
|  |  | MSX1 | 2,82E-56 | GFI1 | 1,32E-38 |
|  |  | IRF2 | 3,28E-55 | FOXO3 | 3,64E-38 |
|  |  | MEIS1AHOXA9 | 6,85E-55 | MSX1 | 4,87E-38 |
|  |  | TST1 | 2,46E-54 | CEBPA | 3,88E-37 |
|  |  | CREBP1 | 1,00E-53 | TST1 | 1,20E-36 |
|  |  | MRF2 | 1,51E-53 | FAC1 | 1,41E-36 |
|  |  | GFI1 | 3,29E-53 | IRF7 | 3,29E-35 |
|  |  | TGIF | 1,60E-51 | STAT5B | 1,93E-34 |
|  |  | GATA6 | 2,80E-51 | RORA2 | 2,84E-33 |
|  |  | IRF7 | 4,99E-51 | ISRE | 2,95E-33 |
|  |  | CEBPB | 1,26E-50 | FREAC2 | 7,32E-33 |
|  |  | okt.01 | 3,86E-47 | GATA1 | 1,07E-32 |
|  |  | EN1 | 5,47E-47 | MEIS1AHOXA9 | 5,53E-31 |
|  |  | RORA1 | 5,52E-45 | AML1 | 1,57E-29 |
|  |  | HOXA3 | 5,33E-44 | BACH1 | 9,95E-29 |
|  |  | PAX6 | 7,17E-43 | EN1 | 4,42E-28 |
|  |  | RP58 | 4,59E-41 | PAX6 | 1,06E-27 |
|  |  | FAC1 | 5,16E-41 | MRF2 | 1,19E-26 |
|  |  | STAT | 1,15E-38 | HSF2 | 9,57E-26 |
|  |  | ISRE | 1,06E-36 | TGIF | 8,76E-25 |
|  |  | CDPCR1 | 8,55E-36 | STAT | 1,23E-24 |
|  |  | MEIS1 | 1,82E-35 | HOXA3 | 4,34E-24 |
|  |  | TAL1BETAITF2 | 1,96E-34 | MYB | 2,18E-23 |
|  |  | CEBP | 5,10E-33 | SRF | 3,94E-23 |
|  |  | CDPCR3 | 6,50E-31 | AP1 | 8,02E-23 |
|  |  | AP1 | 3,33E-30 | RP58 | 9,87E-23 |
|  |  | BACH1 | 2,30E-29 | PAX4 | 1,31E-22 |
|  |  | BRACH | 3,19E-29 | GATA6 | 9,58E-22 |
|  |  | HSF2 | 3,64E-29 | BRACH | 1,03E-21 |
|  |  | SRF | 3,42E-28 | CDPCR3 | 1,42E-20 |
|  |  | HSF1 | 4,09E-28 | HSF1 | 2,99E-20 |
|  |  | CDPCR3HD | 2,88E-27 | MEIS1 | 3,50E-19 |
|  |  | GATA1 | 8,15E-27 | IK3 | 6,43E-19 |
|  |  | AML1 | 9,29E-26 | HAND1E47 | 4,91E-18 |
|  |  | HAND1E47 | 2,25E-25 | CHOP | 1,16E-17 |
|  |  | TAL1BETAE47 | 5,94E-24 | RFX1 | 1,47E-16 |
|  |  | TCF11 | 2,61E-20 | LYF1 | 3,07E-16 |
|  |  | IK1 | 5,50E-20 | BACH2 | 4,32E-16 |
|  |  | NFE2 | 1,95E-19 | IK1 | 4,62E-16 |
|  |  | TCF11MAFG | 4,63E-19 | CDPCR1 | 4,67E-16 |
|  |  | CHOP | 9,87E-19 | NFKAPPAB65 | 6,56E-16 |
|  |  | BACH2 | 2,69E-18 | RORA1 | 6,68E-15 |
|  |  | IK3 | 1,62E-17 | STAT5A | 1,42E-14 |
|  |  | MYB | 2,43E-17 | NFY | 6,04E-14 |
|  |  | STAT5A | 3,00E-16 | PAX2 | 1,51E-13 |
|  |  | LMO2COM | 3,11E-16 | IK2 | 1,81E-13 |
|  |  | NCX | 4,92E-16 | CDPCR3HD | 4,21E-13 |
|  |  | PAX4 | 1,01E-15 | NCX | 4,69E-13 |
|  |  | AP1FJ | 1,69E-15 | NFE2 | 3,04E-12 |
|  |  | HTF | 3,04E-15 | HTF | 5,29E-12 |
|  |  | TAL1ALPHAE47 | 1,08E-13 | AREB6 | 9,71E-12 |
|  |  | IK2 | 3,85E-13 | GCNF | 4,82E-11 |
|  |  | NFKAPPAB65 | 4,12E-12 | GRE | 6,99E-11 |
|  |  | LYF1 | 1,97E-11 | TAL1BETAITF2 | 1,18E-10 |
|  |  | RFX1 | 6,08E-11 | STAT1 | 2,23E-10 |
|  |  | GR | 1,18E-10 | E2F | 2,90E-10 |
|  |  | GRE | 1,50E-10 | TCF11 | 3,24E-10 |
|  |  | STAT1 | 3,27E-10 | TCF11MAFG | 6,57E-10 |
|  |  | GCNF | 2,56E-09 | STAT3 | 1,44E-09 |
|  |  | CREBP1CJUN | 7,15E-09 | GATA3 | 1,55E-09 |
|  |  | NFY | 1,05E-08 | P53 | 2,55E-09 |
|  |  | COUP | 1,13E-08 | SREBP1 | 1,05E-08 |
|  |  | PAX3 | 1,23E-08 | COUP | 1,13E-08 |
|  |  | CREL | 1,91E-07 | PAX3 | 1,38E-08 |
|  |  | STAT3 | 2,11E-07 | YY1 | 2,78E-08 |
|  |  | NF1 | 2,83E-07 | USF | 6,04E-08 |
|  |  | HMX1 | 4,42E-07 | GR | 9,98E-08 |
|  |  | PPARG | 4,95E-07 | AP2REP | 1,24E-07 |
|  |  | PPARA | 1,38E-06 | PPARG | 1,70E-07 |
|  |  | MAX | 2,45E-06 | LUN1 | 2,30E-07 |
|  |  | GATA3 | 4,90E-06 | ATF6 | 3,08E-07 |
|  |  | E2F | 4,31E-05 | CREBP1CJUN | 4,32E-07 |
|  |  | SREBP1 | 5,55E-05 | HMX1 | 4,93E-07 |
|  |  | EGR2 | 1,23E-04 | LMO2COM | 8,78E-07 |
|  |  | SEF1 | 2,30E-04 | NF1 | 3,19E-06 |
|  |  | PAX2 | 2,54E-04 | CREL | 4,57E-06 |
|  |  | COMP1 | 4,22E-04 | SEF1 | 5,38E-06 |
|  |  | YY1 | 4,50E-04 | CREB | 7,36E-06 |
|  |  | AREB6 | 4,71E-04 | COMP1 | 9,72E-06 |
|  |  |  |  | NFKAPPAB | 2,28E-05 |
|  |  |  |  | MYCMAX | 2,95E-05 |
|  |  |  |  | TAL1ALPHAE47 | 3,87E-05 |
|  |  |  |  | NFKB | 4,97E-05 |
|  |  |  |  | E47 | 5,08E-05 |
|  |  |  |  | TAL1BETAE47 | 6,00E-05 |
|  |  |  |  | AP1FJ | 7,20E-05 |
|  |  |  |  | MZF1 | 1,78E-04 |
|  |  |  |  | ER | 2,92E-04 |
|  |  |  |  | XBP1 | 5,15E-04 |
|  |  |  |  | HOX13 | 6,83E-04 |

**STable 3. Pscan - Transcription Factor Binding Site enrichment in gene promoters associated to respective core type genomic bins**

Enrichment analysis of TFBSs associated with gene promoters (-200bp and +50bp to TSS) of core type genomic bins was done using Pscan tools (*http://159.149.160.88/pscan/*). Binding site data is sourced from JASPAR. Results shown are filtered for P-value <0.001. The results are purely computational and not all binding sites listed here are biologically functional binding sites.

| Type I |  | Type II |  | Type III |  |
| --- | --- | --- | --- | --- | --- |
| TF | **P_VALUE** | **TF** | **P_VALUE** | **TF** | **P_VALUE** |
| NRF1 | 2.69E-05 | E2F4 | 1.04E-06 | E2F4 | 2.41E-84 |
| EGR1 | 0.000405 | Tcfl5 | 9.22E-06 | SP2 | 9.24E-83 |
| EGR3 | 0.000441 | ZNF263 | 1.80E-05 | NRF1 | 3.34E-80 |
| HINFP | 0.000682 | EGR3 | 3.87E-05 | TFDP1 | 2.74E-74 |
| Hes1 | 0.000702 | E2F6 | 7.54E-05 | SP1 | 3.48E-72 |
| EGR4 | 0.000805 | ZBTB33 | 0.000143 | EGR3 | 8.84E-70 |
| SP3 | 0.000885 | EGR1 | 0.000161 | Tcfl5 | 1.24E-69 |
| ARNT::HIF1A | 0.00095 | TFDP1 | 0.000461 | EGR1 | 4.92E-63 |
|  |  | EGR2 | 0.000993 | KLF16 | 4.94E-63 |
|  |  |  |  | EGR2 | 4.23E-62 |
|  |  |  |  | SP3 | 8.42E-60 |
|  |  |  |  | KLF5 | 5.16E-58 |
|  |  |  |  | HINFP | 5.19E-58 |
|  |  |  |  | KLF14 | 3.36E-57 |
|  |  |  |  | EGR4 | 1.65E-53 |
|  |  |  |  | SP4 | 7.70E-52 |
|  |  |  |  | SP8 | 2.78E-50 |
|  |  |  |  | E2F6 | 4.86E-48 |
|  |  |  |  | Hes1 | 3.75E-46 |
|  |  |  |  | CTCFL | 4.43E-46 |
|  |  |  |  | ZBTB33 | 3.37E-42 |
|  |  |  |  | TFAP2A | 1.46E-37 |
|  |  |  |  | ZIC3 | 8.74E-36 |
|  |  |  |  | HEY1 | 6.71E-35 |
|  |  |  |  | Gmeb1 | 3.89E-34 |
|  |  |  |  | ZNF740 | 1.08E-33 |
|  |  |  |  | ZIC4 | 2.13E-29 |
|  |  |  |  | TFAP2B(var.3) | 3.76E-29 |
|  |  |  |  | ZIC1 | 1.11E-28 |
|  |  |  |  | GLIS2 | 1.39E-28 |
|  |  |  |  | Zfx | 3.18E-27 |
|  |  |  |  | E2F1 | 3.64E-27 |
|  |  |  |  | TFAP2C | 6.83E-27 |
|  |  |  |  | PLAG1 | 4.44E-26 |
|  |  |  |  | Klf12 | 2.80E-25 |
|  |  |  |  | ZBTB7A | 5.25E-25 |
|  |  |  |  | HEY2 | 6.65E-25 |
|  |  |  |  | HES7 | 1.23E-24 |
|  |  |  |  | ARNT::HIF1A | 1.99E-24 |
|  |  |  |  | PAX5 | 6.01E-24 |
|  |  |  |  | HIF1A | 2.43E-22 |
|  |  |  |  | HES5 | 1.21E-21 |
|  |  |  |  | Gabpa | 4.26E-21 |
|  |  |  |  | CENPB | 2.18E-20 |
|  |  |  |  | Klf1 | 2.33E-20 |
|  |  |  |  | ZBTB7B | 5.94E-19 |
|  |  |  |  | ZNF263 | 1.40E-18 |
|  |  |  |  | KLF9 | 1.59E-18 |
|  |  |  |  | Ahr::Arnt | 2.48E-17 |
|  |  |  |  | KLF13 | 7.03E-16 |
|  |  |  |  | ELK4 | 1.12E-14 |
|  |  |  |  | GLIS1 | 6.10E-14 |
|  |  |  |  | MZF1(var.2) | 6.41E-14 |
|  |  |  |  | THAP1 | 1.07E-13 |
|  |  |  |  | MXI1 | 1.15E-13 |
|  |  |  |  | ZBTB7C | 3.59E-13 |
|  |  |  |  | ELK1 | 3.81E-13 |
|  |  |  |  | ETV5 | 4.92E-13 |
|  |  |  |  | BHLHE40 | 2.53E-12 |
|  |  |  |  | INSM1 | 3.44E-12 |
|  |  |  |  | MYCN | 1.38E-11 |
|  |  |  |  | MYC | 1.33E-10 |
|  |  |  |  | CTCF | 1.87E-10 |
|  |  |  |  | ETV6 | 3.33E-09 |
|  |  |  |  | ASCL1 | 4.55E-09 |
|  |  |  |  | NHLH1 | 4.64E-09 |
|  |  |  |  | ETV1 | 1.12E-08 |
|  |  |  |  | NR2C2 | 1.34E-08 |
|  |  |  |  | ELK3 | 4.98E-08 |
|  |  |  |  | BHLHE41 | 1.22E-07 |
|  |  |  |  | ELF4 | 2.06E-07 |
|  |  |  |  | Arnt | 4.86E-07 |
|  |  |  |  | GLI2 | 8.61E-07 |
|  |  |  |  | GCM2 | 9.12E-07 |
|  |  |  |  | NFKB1 | 3.23E-06 |
|  |  |  |  | ETV4 | 3.61E-06 |
|  |  |  |  | MTF1 | 3.82E-06 |
|  |  |  |  | PAX9 | 5.51E-06 |
|  |  |  |  | NFKB2 | 6.27E-06 |
|  |  |  |  | Arntl | 7.50E-06 |
|  |  |  |  | ELF1 | 1.66E-05 |
|  |  |  |  | PAX1 | 2.07E-05 |
|  |  |  |  | GCM1 | 2.90E-05 |
|  |  |  |  | FLI1 | 4.73E-05 |
|  |  |  |  | ERF | 5.44E-05 |
|  |  |  |  | GLIS3 | 0.000114 |
|  |  |  |  | RREB1 | 0.000152 |
|  |  |  |  | FEV | 0.000195 |
|  |  |  |  | EWSR1-FLI1 | 0.000228 |
|  |  |  |  | CREB3 | 0.000303 |
|  |  |  |  | Creb3l2 | 0.000307 |
|  |  |  |  | ETS1 | 0.00037 |
|  |  |  |  | CREB3L1 | 0.000542 |
|  |  |  |  | Atf1 | 0.000671 |

**STable 4. PASTAA - Transcription Factor Binding Site enrichment in gene promoters associated to respective core type genomic bins**

Enrichment analysis of TFBSs associated with gene promoters (-200bp to TSS) of core type genomic bins was done using PASTAA tools *(http://trap.molgen.mpg.de/PASTAA.htm*). Binding site data is sourced from TRANSFAC. Results shown are filtered for P-value <0.001. The results are purely computational and not all binding sites listed here are biologically functional binding sites.

| Type I |  | Type II |  | Type III |  |
| --- | --- | --- | --- | --- | --- |
| TF | **P-Value** | **TF** | **P-Value** | **TF** | **P-Value** |
| E2F | 0 | ZF5 | 0 | ZF5 | 0 |
| AP2ALPHA | 0 | HIC1 | 0 | CREB | 0 |
| ZF5 | 0 | SPZ1 | 1.20E-05 | CETS1P54 | 0 |
| KROX | 0 | E2F | 5.30E-05 | E2F | 0 |
| CHCH | 0 | CREB | 0.000134 | EGR1 | 1.00E-06 |
| MAZ | 2.00E-06 | HES1 | 0.000156 | MAZR | 4.00E-06 |
| AP2GAMMA | 5.00E-06 | TFIII | 0.000188 | HIF1 | 1.50E-05 |
| CREB | 9.40E-05 | MYCMAX | 0.000494 | ATF3 | 3.60E-05 |
| SP1 | 0.000126 | ATF3 | 0.00056 | CREBP1 | 6.10E-05 |
| VJUN | 0.000256 | E2F1 | 0.000849 | HIC1 | 6.40E-05 |
| BLIMP1 | 0.00041 | AMEF2 | 0.000946 | MAZ | 0.000119 |
| MOVOB | 0.000761 | CHCH | 0.00096 | E2F1 | 0.000122 |
|  |  |  |  | CREBATF | 0.000296 |
|  |  |  |  | VJUN | 0.00039 |
|  |  |  |  | NRF1 | 0.000483 |
|  |  |  |  | AHRHIF | 0.000521 |
|  |  |  |  | AP2ALPHA | 0.000533 |
|  |  |  |  | NRSE | 0.000533 |
|  |  |  |  | MZF1 | 0.000853 |
|  |  |  |  | WHN | 0.000948 |

**Supplementary References**

1. Zhu, Y., Chen, Z., Zhang, K., et al. (2016) Constructing 3D interaction maps from 1D epigenomes, *Nat Commun* *7*.

2. Heidari, N., Phanstiel, D. H., He, C., et al. (2014) Genome-wide map of regulatory interactions in the human genome, *Genome research* *24*, 1905-1917.

3. Cook, P. R. (2010) A Model for all Genomes: The Role of Transcription Factories, *Journal of molecular biology* *395*, 1-10.
